# Multi-Year Persistence of Verotoxigenic *Escherichia coli* (VTEC) in a Closed Canadian Beef Herd: A Cohort Study

**DOI:** 10.3389/fmicb.2018.02040

**Published:** 2018-08-31

**Authors:** Lu Ya Ruth Wang, Cassandra C. Jokinen, Chad R. Laing, Roger P. Johnson, Kim Ziebell, Victor P. J. Gannon

**Affiliations:** ^1^National Microbiology Laboratory, Public Health Agency of Canada, Lethbridge, AB, Canada; ^2^Alberta Agriculture and Forestry, Lethbridge, AB, Canada; ^3^National Microbiology Laboratory, Public Health Agency of Canada, Guelph, ON, Canada

**Keywords:** VTEC, STEC, cattle, serotype, persistence, virulence, genomics

## Abstract

In this study, fecal samples were collected from a closed beef herd in Alberta, Canada from 2012 to 2015. To limit serotype bias, which was observed in enrichment broth cultures, Verotoxigenic *Escherichia coli* (VTEC) were isolated directly from samples using a hydrophobic grid-membrane filter verotoxin immunoblot assay. Overall VTEC isolation rates were similar for three different cohorts of yearling heifers on both an annual (68.5 to 71.8%) and seasonal basis (67.3 to 76.0%). Across all three cohorts, O139:H19 (37.1% of VTEC-positive samples), O22:H8 (15.8%) and O?(O108):H8 (15.4%) were among the most prevalent serotypes. However, isolation rates for serotypes O139:H19, O130:H38, O6:H34, O91:H21, and O113:H21 differed significantly between cohort-years, as did isolation rates for some serotypes within a single heifer cohort. There was a high level of VTEC serotype diversity with an average of 4.3 serotypes isolated per heifer and 65.8% of the heifers classified as “persistent shedders” of VTEC based on the criteria of >50% of samples positive and ≥4 consecutive samples positive. Only 26.8% (90/336) of the VTEC isolates from yearling heifers belonged to the human disease-associated seropathotypes A (O157:H7), B (O26:H11, O111:NM), and C (O22:H8, O91:H21, O113:H21, O137:H41, O2:H6). Conversely, seropathotypes B (O26:NM, O111:NM) and C (O91:H21, O2:H29) strains were dominant (76.0%, 19/25) among VTEC isolates from month-old calves from this herd. Among VTEC from heifers, carriage rates of *vt1*, *vt2*, *vt1+vt2*, *eae*, and *hlyA* were 10.7, 20.8, 68.5, 3.9, and 88.7%, respectively. The adhesin gene *saa* was present in 82.7% of heifer strains but absent from all of 13 *eae*+ve strains (from serotypes/intimin types O157:H7/γ1, O26:H11/β1, O111:NM/θ, O84:H2/ζ, and O182:H25/ζ). Phylogenetic relationships inferred from wgMLST and pan genome-derived core SNP analysis showed that strains clustered by phylotype and serotype. Further, VTEC strains of the same serotype usually shared the same suite of antibiotic resistance and virulence genes, suggesting the circulation of dominant clones within this distinct herd. This study provides insight into the diverse and dynamic nature of VTEC populations within groups of cattle and points to a broad spectrum of human health risks associated with these *E. coli* strains.

## Introduction

Verotoxigenic *Escherichia coli* (VTEC) are a group of toxin-producing bacteria implicated in gastrointestinal infections and foodborne outbreaks worldwide (Bettelheim, 2007). Public health surveillance efforts have prioritized serotype O157:H7 due to its relative impact on the human disease burden – including its propensity to cause severe illness and outbreaks (Bettelheim, 2007; Gyles, 2007; CDC, 2012, 2018), the ease of laboratory identification (March and Ratnam, 1986), and the relative paucity of detailed clinical data on non-O157 related haemolytic uremic syndrome (HUS) (Tarr et al., 2005). However, non-O157 serotypes account for a significant proportion of VTEC isolated from both sporadic cases and outbreaks of clinical diarrheal disease (Wang et al., 2005, 2013; Buvens et al., 2012; EFSA, 2013; Momtaz et al., 2013; Bettelheim and Goldwater, 2014). Non-O157 VTEC have been isolated from many sources including, environmental and drinking water (Johnson et al., 2014), flour (Morton et al., 2017), lettuce (Taylor et al., 2013), unpasteurized cheese and apple juice (Schaffzin et al., 2012; Guzman-Hernandez et al., 2016), meat (Bosilevac and Koohmaraie, 2011; Momtaz et al., 2013), bovine carcasses and hides (Arthur et al., 2002; Barkocy-Gallagher et al., 2003; Monaghan et al., 2012), and cattle feces (Hussein and Bollinger, 2005; Monaghan et al., 2011).

While cattle are recognized as important hosts for human VTEC infections (Caprioli et al., 2005; Bettelheim and Goldwater, 2014), knowledge gaps in the ecology of VTEC persist. For example, cattle density per unit area was positively correlated with an increased risk of human infection, but not for clinically associated serogroups O26 and O91 (Frank et al., 2008). Clinical cases of VTEC O157 have been shown to peak during the summer and fall months (Tarr et al., 2005; CDC, 2012), coinciding with elevated shedding of the organisms in cattle feces (Van Donkersgoed et al., 1999; Blanco et al., 2008). However, divergent seasonal shedding patterns in cattle have been reported for non-O157 serotypes (Barkocy-Gallagher et al., 2003) and whether any seasonality exists for non-O157 VTEC disease incidence remains unclear (Klein et al., 2002; Tarr et al., 2005; CDC, 2012; Rivero et al., 2012). Even for O157, seasonal trends have not been observed in all studies (Alam and Zurek, 2006; Ellis-Iversen et al., 2009; Lammers et al., 2015).

Several longitudinal studies have examined the persistence of VTEC within cattle herds. However, the majority have focused on O157 or other top clinical serotypes (Smith et al., 2010; Joris et al., 2013; Dewsbury et al., 2015; Lammers et al., 2015). Other studies sampled cattle of varying ages with fewer animals being sampled for each cohort or were focused on calves (Cobbold and Desmarchelier, 2000; Pearce et al., 2004). Few studies were inclusive of all VTEC serotypes, or monitored a particular herd regularly over a period of more than 12 months (Geue et al., 2002); many utilized pooled samples, pen-floor samples, random sampling of multiple herds, transient cattle populations, infrequent or seasonal sampling, and/or PCR pre-screening, with significantly lower isolation rates (Jenkins et al., 2002; Monaghan et al., 2011; Joris et al., 2013; Dewsbury et al., 2015; Widgren et al., 2015; Stanford et al., 2016). While these studies offer indispensable and comprehensive examinations of VTEC in cattle, we felt it would be beneficial to narrow the scope and focus on the herd level, with individual sampling of a specific group of cattle over multiple years using culture-based procedures which do not select for specific VTEC serotypes. In this way, confounding factors such as differences in management practice and the age may be minimized, while elucidating patterns of multi-year persistence for certain serotypes as well as the intra-cohort contribution of individuals to serotype prevalence.

In this study, three consecutive cohorts of yearling heifers within a closed beef herd were monitored for VTEC using a hydrophobic grid-membrane filter (HGMF) verotoxin immunoblot method. Isolates were characterized by serotype, phylotype, and virulence-associated and antibiotic resistance gene content. Phylogenetic relationships and diversity were explored using whole genome sequence data. The objective was to examine the diversity of VTEC within a specific herd over time and the associated human health risks.

## Materials and Methods

### Herd Characteristics and Sample Collection

This study was carried out in accordance with the principles contained in the “Guide to the Care and Use of Experimental Animals,” Vols. I and II, by the Canadian Council on Animal Care (CCAC) and followed CCAC Guidelines. The protocol was approved by the Canadian Food Inspection Agency National Centre for Animal Disease (CFIA-NCAD) Animal Care Committee (protocol # 1201).

Three consecutive cohorts of yearling heifers (*n* = 10, 12, and 16) from the CFIA-NCAD (Lethbridge, AB, Canada) Angus-Hereford cross research herd were sampled monthly from April 2012 to March 2013, April 2013 to March 2014 and May 2014 to March 2015. The closed herd has a size and management system similar to that found in beef cow-calf herds in western Canada, has been previously sampled for *E. coli* O157:H7 (Gannon et al., 2002), and shares high genetic homogeneity due to its restricted repopulation. A total of 368 samples were collected; 103, 111 and 154 in each year, respectively. Fecal samples were collected by rectal palpation, except in July and August 2012 and August 2014 when samples were collected fresh from the ground after defecation was observed. A single event sampling of calves born from 7, 6, and 15 of the sampled heifers was conducted approximately 1 month after parturition in the spring of 2013, 2014, and 2015, respectively.

### Detection and Isolation of VTEC

Methods used for the detection and isolation of VTEC were similar to those described in Atalla et al. (2000), Karama et al. (2008), Johnson et al. (2014), Nadya et al. (2016), and Falardeau et al. (2017) with modifications for unenriched fecal samples.

#### Preparation of VT-Immunoblot (VT-IB) Membranes and VT-ELISA Plates

Capture membranes (82 mm nitrocellulose membranes, pore size 0.2 mm, BioTrace, Pall Life Sciences, Mississauga, ON, Canada) were coated with rabbit anti-VT antibodies reactive to all known verotoxins (PHAC NML, Guelph, ON, Canada) diluted to 2 μg/mL in carbonate:bicarbonate buffer (Sigma, Oakville, ON, Canada) by shaking overnight at 4°C and blocked with a post-coat solution (20% sucrose, 2% Bovine Albumin Fraction V, MP Biomedicals, Santa Ana, CA, United States). Membranes were stored at 4°C with a humidity sponge desiccant (Fisher Scientific, Ottawa, ON, Canada) until use. Immediately before use, membranes were washed (PBS, 0.1% Tween 20) and laid on modified Tryptic Soy Agar (Oxoid, Nepean, ON, Canada) supplemented with 1.5 g/L bile salts No. 3, 10 μg/mL vancomycin, 10 μg/mL cefsulodin and 100 mg/L 5-bromo-4 chloro-3-indoyl-ß-D-glucuronide (Sigma) (mTSAVC-BCIG). To prepare VT-ELISA plates, 50 μL of rabbit anti-VT antibodies diluted to 2 μg/mL in carbonate: bicarbonate buffer (Sigma) was used to coat each well of a 96-well ELISA plate (Nunc-Immuno Loose Well Modules, VWR, Mississauga, ON, Canada). Plates were covered with sealing tape, incubated at 37°C for 1 h, and at 4°C overnight. The post-coat solution was prepared and stirred overnight at 4°C. The post-coat solution and plates were warmed to 37°C for an hour and 150 μL of the post-coat solution was added to each well, followed by incubation at 37°C for 1 h. Post-incubation, the contents of the wells were discarded and the plates dried under a clean biosafety cabinet for 3–4 h. Coated plates were covered with sealing tape and stored at 4°C with a desiccant until use. Immediately before use, plates were equilibrated to room temperature.

#### HGMF Filtration of Unenriched Cattle Fecal Slurry on mTSAVC-BCIG Media

Ten grams of feces were homogenized in 10 mL Phosphate Buffer Saline (PBS). For each sample, 0.01 and 0.02 g of the 1:1 slurry, representing 0.005 and 0.01 g of fecal sample, respectively, were weighed and added to 8 mL aliquots of wash buffer (PBS, 0.1% Tween 20). Between May-August 2012, 0.01 and 0.005 g of unsuspended fecal samples were weighed and added to the wash buffer. The entire 8 mL volumes of both sample dilutions were filtered through sterile 0.45 μM HGMF ISO-GRID filters (Neogen, Lansing, MI, United States) using a HGMF Spreadfilter (FILTAFLEX, Ltd., Almonte, ON, Canada). Positive (isolate EC19920459; bovine O163:NM *vt1*+ *vt2*+) (PHAC NML Guelph) and negative (ATCC *E. coli* 25922; *vt*-) controls were streaked directly to the sterile HGMF ISO-GRID filter. Filters were overlaid on coated capture membranes and the mTSAVC-BCIG agar plates were incubated inverted at 37°C for 18–24 h.

#### Capture Membrane Development, VT-ELISA, and PCR Confirmation of *vt*

VT-capture membrane and HGMF grid filter assemblies were marked by needle punctures for re-orientation. The HGMFs were reserved and the VT-capture membrane was separated from the plate and probed with a mixture of four monoclonal antibodies (each at 2 μg/mL), (PHAC NML Guelph) followed by alkaline phosphatase-labeled rabbit anti-mouse IgG (0.6 mg/mL) (Jackson Immunoresearch, Cedarlane Laboratories, Burlington, ON, Canada) and substrates nitroblue tetrazolium and 5-bromo-4-choloro-3-indolyl-phosphate (Sigma) with 30 min incubations (10 min for final development) and 10 min washes (PBS, 0.1% Tween 20). Verotoxin producing colonies on the HGMFs corresponding in location to purple spots on the VT-immunoblot were picked and inoculated into 800 μL of modified Tryptic Soy Broth supplemented with 1.5 g/L bile salts No. 3, 10 μg/mL vancomycin and 10 μg/mL cefsulodin (Sigma) (mTSBVC) and incubated at 37°C for 18–24 h. The VT-ELISA was performed on duplicate 50 μL aliquots of the overnight broth. Briefly, aliquots of the broth were added to the wells for 30 min then probed sequentially with 50 μL of a mixture of four monoclonal antibodies (2 μg/mL) (PHAC NML Guelph), horseradish-peroxidase-labeled rabbit anti-mouse IgG (0.2 μg/mL) (Jackson Immunoresearch, Cedarlane Laboratories) with 30 min incubations and 5 × 300 μL washes (PBS, 0.1% Tween 20) following each addition using the Asys Atlantis 2 microplate washer (Biochrom, Cambridge, United Kingdom). Plates were developed with 50 μL of substrate tetramethylbenzidine (Sigma) for 10 min with slow agitation, followed by 50 μL of 0.2 M sulfuric acid. Absorbance readings were measured immediately using the μQuant microplate spectrophotometer (BioTek, Winooski, VT, United States) at OD450 nm and 600 nm and the final reading was calculated by subtracting the reading at 600 nm from the reading at 450 nm and averaging the duplicate values. Verotoxin production was scored as positive if OD readings were >2× the mean of the negative toxin control and negative if <1.5× that of the negative control. Suspect broths were streaked for single colonies to mTSAVC-BCIG and incubated at 37°C for 18–24 h. Based on the VT-ELISA OD reading, 3 to 9 colonies of varying morphologies from each test sample were inoculated into mTSBVC and incubated at 37°C for 18–24 h. A secondary VT-ELISA was performed as above and presumptive isolates were streaked to a MacConkey agar lawn for DNA extraction using the EZ1 DNA Tissue Kit (Qiagen, Hilden, Germany) and the EZ1 Advanced XL or the BioRobot EZ1 (Qiagen) and assayed for the presence of *vt1* and *vt2* using PCR (Gannon et al., 1997). DNA and 30% glycerol stocks were stored at –70°C.

#### Comparison of VTEC Isolation From Enriched and Unenriched Fecal Samples

Johnson et al. (2014) demonstrated the efficacy of the VT-IB without enrichment for environmental water samples. In this study, a subset of samples was used to compare the efficacy of testing unenriched and enriched cattle fecal samples. Briefly, for the enriched protocol, the 1:1 fecal slurry was inoculated into 15 mL of mTSBVC and incubated at 37°C for 18–24 h. Overnight broths (2 × 50 μL) were screened by VT-ELISA and based on the OD readings, 100 μL of up to three 10-fold dilutions ranging from 10^-2^ to 10^-7^ were suspended in 10 mL of wash buffer and filtered as per the direct method. A total of 64 samples with at least one successful isolate from either method were used for method comparison. 10/64 samples were also selected for in-depth sampling for serotype diversity by picking at least five colonies per method. Enrichment data was used for method validation only and not carried forward for prevalence studies.

#### Detection of *vt1*, *vt2*, *eae*, *hlyA*, *saa*, and Serotyping

PCR confirmation of *vt1*, *vt2*, *eae*, and *hlyA* (Paton and Paton, 1998), and O:H serotyping were completed by the Public Health Agency of Canada, National Microbiology Laboratory, at the *E. coli* Reference Laboratory in Guelph, Ontario. PCR screening for *vt2* subtypes *a*, *c*, *d* was completed in-house as per (Scheutz et al., 2012) with the following parameters: initial denaturation 95°C, 35× [94°C for 50 s, 64°C for 40 s, 72°C for 60 s], final extension 72°C for 3 min, hold 4°C; 25 μL reactions [1 μL gDNA, 200 nM primers, 1× HotStarTaq Master Mix Kit (Qiagen), 0.2 mM dNTPs]. Confirmatory PCRs for *vt2c* and *vt2d* were completed at an annealing temperature of 66 and 62°C, respectively. PCR screening for *saa* was completed in-house (Paton et al., 2001) with the following parameters: initial denaturation 94°C, 30× [94°C for 15 s, 64°C for 15 s, 72°C for 75 s], final extension 72°C for 5 min, hold 4°C; 25 μL reactions [1 μL gDNA; 200 nM primers, 1.5 mM MgCl_2_, 0.2 mM dNTPs, 2.5 U/rxn Taq (MP Biomedicals, Santa Ana, CA, United States)]. Primer sequences are as listed in **Supplementary Table 1**. Seropathotype (SPT) classifications were assigned based on the Karmali scheme (Karmali et al., 2003). For serotypes not found in the original Karmali scheme, a modified SPT was used (EFSA, 2013) or otherwise assigned by the authors based on the literature, e.g., reported cases of HUS.

### Whole Genome Sequencing and Analysis

One hundred and seventy-nine isolates spanning all serotypes were selected for whole genome sequencing. Genomic DNA was extracted from pure cultures using the MasterPure DNA Purification Kit (Epicentre, Madison, WI, United States) or the DNeasy Blood and Tissue Kits (Qiagen). 2 × 300 bp paired-end sequencing was performed on an Illumina MiSeq (Illumina, San Diego, CA, United States) using Nextera XT libraries and the MiSeq Reagent Kit v3 600 cycles (Illumina). Raw sequencing data has been deposited in the Sequence Read Archive (SRA) at the National Center for Biotechnology Information (NCBI) under BioProject PRJNA473796. Paired-end read data for 72 reference strains for serotypes isolated in this study were downloaded from the NCBI-SRA. A complete list of strains and corresponding meta data is available in **Supplementary Table 10**. *De novo* assembly was performed using the Shovill pipeline (v0.8.0)^1^ which encompasses genome size estimation, FASTQ subsampling, adaptor trimming, read correction, merging of paired-end reads, genome assembly and polishing using Seqtk^2^, KMC 2 (Deorowicz et al., 2015), Trimmomatic (Bolger et al., 2014), Lighter (Song et al., 2014), FLASH (Magoc and Salzberg, 2011), SPAdes (Bankevich et al., 2012), SAMtools (Li et al., 2009), BWA MEM (Li, 2013), and Pilon (Walker et al., 2014).

#### *In silico* Clermont Phylotyping and Whole Genome Based Cluster Analysis

DNA coding sequences for the *arpA*, *chuA*, *yjaA*, and *TspEF.C2* genes (Clermont et al., 2013) were downloaded from NCBI GenBank and queried against a BLAST (v2.2.28) database generated from the 179 draft genomes. Phylogroup was assigned by scoring the presence/absence of the four markers (≥85% sequence identity, ≥60% query length). Cluster analysis was performed and visualized using the goeBURST Full MST algorithm in Phyloviz2 (Nascimento et al., 2017).

Analysis for SNP discovery was performed using Panseq (v3.1.1)^3^ (Laing et al., 2010), with the following parameters: ‘fragmentationSize’ = 500, ‘percentIdentityCutoff’ = ‘85,’ ‘coreGenomeThreshold’ = ‘251,’ ‘runMode’ = ‘pan,’ ‘cdhit’ = ‘1’ and a maximum-likelihood phylogeny was constructed using PhyML (v3.0)^4^ (Guindon et al., 2005), with default parameters (substitution model selection criterion: AIC, type of tree improvement: NNI, branch support: aLRT SH-like). Phylogenetic visualizations were performed using iTOL (v3.5.4)^5^ (Letunic and Bork, 2007). Allele calls from 8,162/17,380 loci using both assembly-free and assembly-based approaches for whole genome MLST (wgMLST) were made for 251 isolates (179 from this study, 72 from NCBI SRA) using Bionumerics v7.6.2. The minimum spanning tree (MST) was constructed in the visualization program GrapeTree using the MSTreeV2 method, which correctly accounts for missing data (Zhou et al., 2018). The Phylotyper program (Whiteside et al., 2017) was used for *in silico* serotyping of draft assemblies (% ID 90, % coverage 95).

#### *In silico* Detection of Virulence-Associated and Antibiotic Resistance Genes

Draft assemblies for a subset (*n* = 115) of project and reference strains spanning all serotypes from this study – including, for each serotype, up to 6 strains from the study and up to 3 reference strains were interrogated for the presence of virulence-associated genes using Panseq (v.3.1.1) (Laing et al., 2010) with a curated list (*n* = 2,710) of virulence-associated gene sequences, including gene variants^6^, with the following parameters: ‘percentIdentityCutoff’ = ‘85,’ ‘coreGenomeThreshold’ = ‘115.’ Gene presence/absence was confirmed in ABRicate v0.7^7^ using the VFDB database (2,597 sequences), with the following parameters: % coverage ≥60, % identity ≥80. The same dataset was further interrogated for the presence of antibiotic resistance genes in ABRicate v0.7 using the ResFinder database (2,228 sequences) and the CARD database (2,153 sequences), with the following parameters: % coverage ≥60, % identity ≥80. Gene presence/absence was confirmed using the RGI tool v4.0.3 (Jia et al., 2017) under “perfect” and “strict” match conditions. The presence/absence data was overlaid onto the core SNP phylogenetic tree and visualized using iTOL (Letunic and Bork, 2007).

#### *In silico* Analysis of *eae* and *hlyA* Genetic Diversity

DNA coding sequence for EHEC-*hlyA* (GenBank Accession X94129.1) and all variants of *eae* from a curated list (*n* = 2,710) of virulence-associated gene sequences^6^ were queried against all study and reference strains (*n* = 251) using BLAST (v2.2.28), (% ID 85, % coverage 60). Per strain, the top BLAST hit [% identity × % query coverage] for EHEC-*hlyA* (*n* = 190) and *eae* (*n* = 32), respectively was carried forward for multiple sequence alignment using MAFFT^8^ (Katoh et al., 2017). The alignments were used to generate a maximum-likelihood phylogeny estimated using default parameter in PhyML as above (Guindon et al., 2005) and visualized using iTOL (Letunic and Bork, 2007).

### Statistical Analysis

**FIGURE 1:**
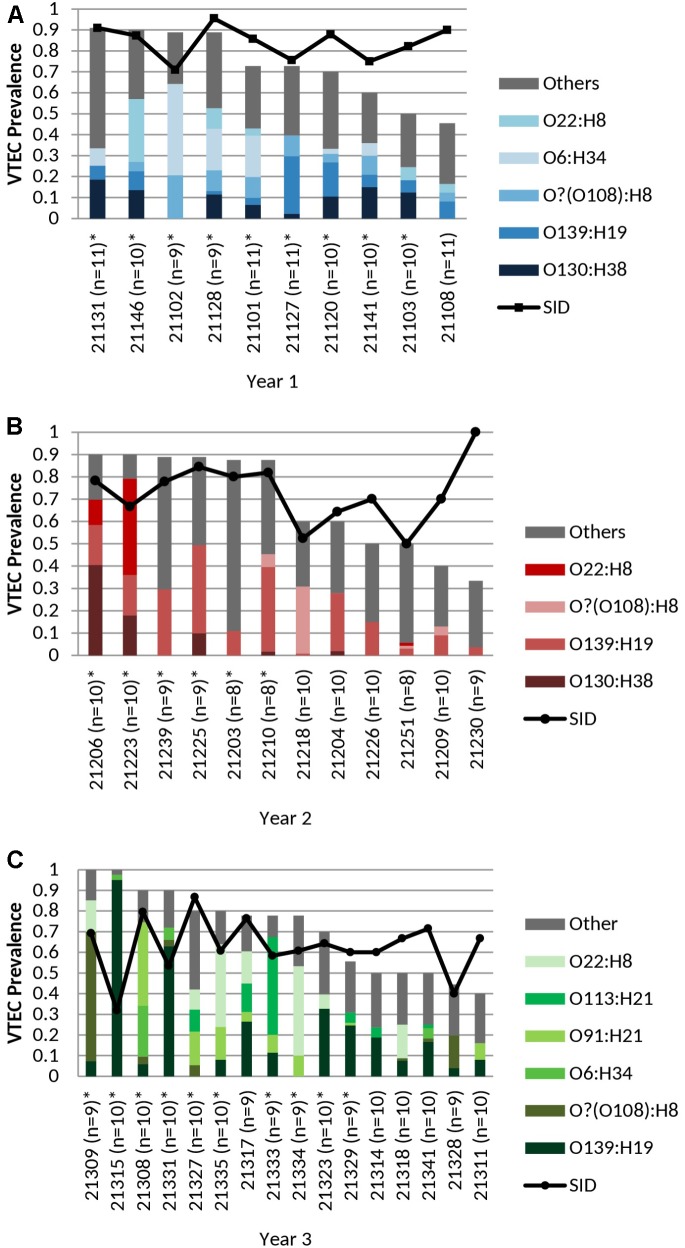
**(A)** Cohort-year 1, **(B)** Cohort-year 2, and **(C)** Cohort-year 3. Cohort-specific VTEC prevalence, serotype distribution, serotype diversity (SID) and persistent shedding in heifers. *n* = no. of samples per animal. Line: Simpson’s Index of Diversity (SID), where 0 = no diversity, 1 = infinite diversity. ^∗^ Persistent shedder (Menrath et al., 2010) (at least half and ≥4 consecutive *vt* positive samples). Bar (total): isolation rate. Bar (stacked colors): serotype distribution of top serotypes (≥5 samples per year). No. of samples: cohort-year 1 (*n* = 103), cohort-year 2 (*n* = 111), and cohort-year 3 (*n* = 154). No. of isolates: cohort-year 1 (*n* = 277), cohort-year 2 (*n* = 283), and cohort-year 3 (*n* = 388). VTEC isolation rates (total) were not statistically different (*p* ≥ 0.05) between individual heifers. Average per-animal VTEC prevalence (isolation) rate: 70.5% (Overall), 72.1% (Year 1), 68.8% (Year 2), 70.8% (Year 3). Average per-animal SID values: 0.72 (Overall), 0.84 (Year 1), 0.73 (Year 2), 0.63 (Year 3).

**FIGURE 2:**
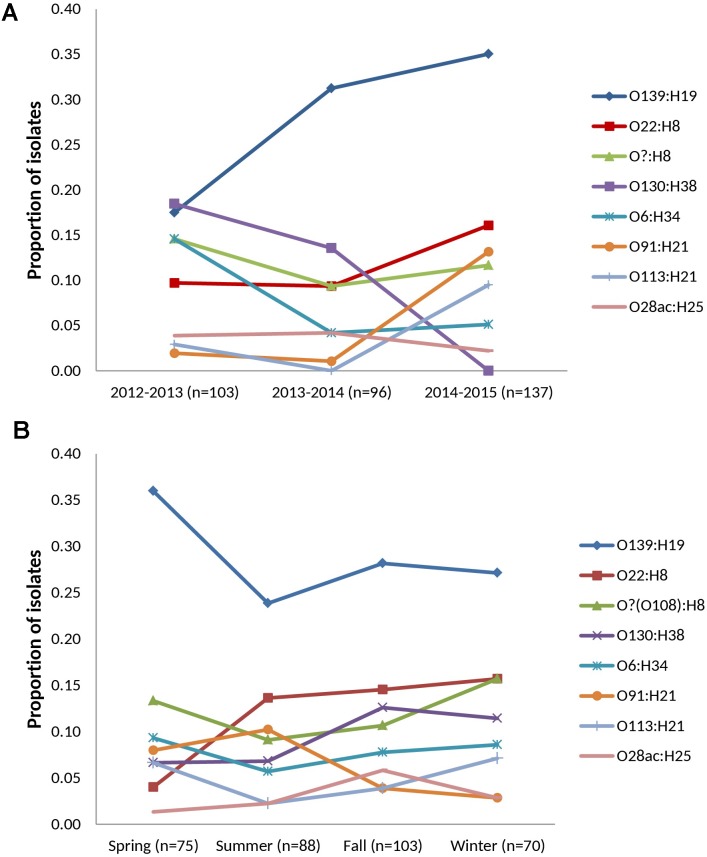
Temporal distribution of prevalent serotypes among heifer isolates. (*n* = 336 strains) **(A)** cohort-year. **(B)** seasonal. Prevalent serotypes (colored lines): serotypes isolated from >10 samples in the study.

A subset of 336 unique strains from the 948 heifer isolates and 25 unique strains from the 79 calf isolates, representing one serotype-virulotype (*vt1*, *vt2*, *hlyA*, *eae*, and *saa*) representative for each fecal sample was carried forward for all analyses unless otherwise stated. Using the R statistical software, the pairwise.fisher.test () function from the {fmsb} package (x, n, p.adjust.methods = “bonferroni”) (α = 0.05) was used for pairwise comparisons of VTEC prevalence. The oddsratio function from the {fmsb} package (a, b, c, d, conf.level = 0.95) was used to calculate the odds ratio and 95% confidence interval for the VT-immunoblot methods comparison. 95% confidence intervals for proportions were calculated using the Wilson procedure with a correction for continuity^9^. Microsoft Excel was used to calculate the standard deviation (STDEV.S) and confidence intervals (CONFIDENCE.T) for the average VTEC isolation rates and average Simpson’s Index of Diversity (SID) values at the herd and cohort levels. SID,

$$1-D=\frac{\Sigma n\left(n-1\right)}{N\left(N-1\right)}$$

where, *n* = number of isolates of a particular serotype and *N* = total number of isolates; 0 = no diversity, 1 = infinite diversity, was used to compare serotype diversity, taking into account the number of serotypes present (“serotype richness”) and the relative abundance (“serotype evenness”). For **Figure 1**, to account for variation in the number of isolates recovered per sample, the contribution of a specific serotype to the serotype distribution in an individual cow was calculated as follows:

for each sample (Y),

$$\frac{\Sigma \left(\frac{\text{no. of isolates of serotype X}}{\text{no. of isolates from sample Y}}\right)}{\text{no. of samples tested from cow Z}}$$

followed by normalization to the isolation rate for that cohort-year. *n* = 277, 283, 388 for years 1, 2, and 3, respectively. For **Figure 2**, statistics source data are as in **Supplementary Table 9**.

## Results

### VT-IB of Bacterial Growth From Unenriched Cattle Fecal HGMF Filtrates on mTSAVC-BCIG Media

VTEC were detected in 74.7% (275/368) and isolated from 70.4% (259/368) of cattle fecal slurry samples plated directly onto HGMF filters on mTSAVC-BCIG media; representing an isolate recovery rate for immunoblot positive samples of 94.2% (**Table 1**). Among a subset of 64 samples tested by both unenriched/direct and enriched methods – 60.9% (39/64), 32.8% (21/64), 6.3% (4/64) were positive by both methods, unenriched only and enriched only, respectively. That is, 93.8% (60/64) of samples were VTEC positive by isolation without a selective broth culture enrichment step. VTEC was 7.3 times more likely to be isolated from immunoblot-positive samples using the direct method than the enrichment method (**Table 2**). The number of serotypes isolated from unenriched and enriched cultures of these samples were 18 and 15, respectively; with 11 serotypes shared between the two methods (**Supplementary Table 2**). Further, in-depth sampling of suspect colonies (≥5 colonies per method) showed only one case where the enrichment method recovered a serotype which was not also recovered directly from unenriched samples (**Supplementary Table 3**).

**Table 1:** Summary of VTEC sampling across three cohorts of heifers.

	Year 1 (Apr ’12–Mar ’13)	Year 2 (Apr ’13–Mar ’14)	Year 3 (May ’14–Mar ’15)	Total (2012–2015)
Number of animals	10	12	16	38
Number of samples	103	111	154	368
Detection rate	82 (79.6%)	79 (71.2%)	114 (74.0%)	275 (74.7%)
Isolation rate	74 (71.8%)	76 (68.5%)	109 (70.8%)	259 (70.4%)
Number of isolates	277	283	388	948
Number of serotypes	17	19	14	32
SID^2^	0.88	0.86	0.81	0.87
Clinically^1^	O26:NM(H11) (3)^∗∗^	O157:H7 (4)^∗∗^	O111:NM(H11) (1)^∗∗^	O157:H7 (4)^∗∗^
Significant	O22:H8 (10)	O111:NM (1)^∗∗^	O22:H8 (22)	O26:NM(H11) (3)^∗∗^
Serotypes	O113:H21 (3)	O22:H8 (9)	O91:H21 (17)	O111:NM (2)^∗∗^
(Number of samples)	O91:H21 (2)	O91:H21 (1)	O113:H21 (12)	O22:H8 (41)
	O2:H6 (1)		O137:H41 (2)	O91:H21 (20)
				O113:H21 (15)
				O137:H41 (2)
				O2:H6 (1)
Persistent	O139:H19 (10)	O139:H19 (10)	O139:H19 (10)	O139:H19 (30)
Serotypes	O6:H34 (10)	O22:H8 (8)	O91:H21 (10)	O?(O108):H8 (24)
≥5 months/years	O?(O108):H8 (9)	O130:H38 (8)	O22:H8 (9)	O22:H8 (23)
(Number of months)	O130:H38 (8)	O?(O108):H8 (7)	O?(O108):H8 (8)	
	O22:H8 (6)		O113:H21 (8)	
			O6:H34 (5)	
Prevalent	O130:H38 (19)	O139:H19 (30)	O139:H19 (48)	**O139:H19 (96)**
Serotypes	O139:H19 (18)	O130:H38 (13)	O22:H8 (22)	**O22:H8 (41)**
≥5 sample/years	O?(O108):H8 (15)	O?(O108):H8 (9)	O91:H21 (18)	**O?(O108):H8 (40)**
(Number of samples)	O6:H34 (15)	O22:H8 (9)	O?(O108):H8 (16)	O130:H38 (32)
	O22:H8 (10)		O113:H21 (12)	**O6:H34 (26)**
			O6:H34 (7)	**O91:H21 (21)**
				O113:H21 (16)
				**O28ac:H25 (11)**
				O132:NM(H18) (8)
				O46:H38 (5)


*^*1*^Seropathotypes (SPT) A, B, or C based on Karmali et al., 2003 scheme. ^*2*^Simpson’s Index of Diversity (1-D), where 0 = no diversity, 1 = infinite diversity. ^∗∗^’Top 7′ clinically significant serotype (O26, O45, O103, O111, O121, O145). (O/H type) serotypes resolved by whole genome sequencing. Bolded serotypes isolated in all three cohort-years.*

**Table 2:** Enriched vs. unenriched VT-Immunoblot prevalence and odds ratio.

	Direct NOT enriched	Enriched NOT direct	Both methods
Number of samples^1^	21	4	39
% Samples	32.8%	6.3%	60.9%
	**Direct^2^**	**Enriched^3^**	**Odds**
Isolated	60	43	60/43 = 1.40
Not isolated	4	21	4/21 = 0.19
Totals	64	64	1.40/0.19 = 7.33^4^


*^*1*^Samples tested by both enriched and unenriched VT-immunoblot and for which as least one isolate was recovered by either or both methods (*n* = 64). ^*2*^Direct NOT enriched + both methods. ^*3*^Enriched NOT direct + both methods. ^*4*^95% confidence interval [CI] = 2.35, 22.88; *p* = 0.0001598).*

### VTEC Prevalence

#### Temporal

Overall VTEC isolation rates for 2012–2013, 2013–2014 and 2014–2015 were 71.8% (74/103), 68.5% (76/111) and 70.8% (109/154), respectively. The weighted average isolation rates for spring, summer, fall and winter were 68.9% (51/74), 67.3% (72/107), 70.5% (79/112), and 76.0% (57/75), respectively and the weighted average monthly isolation rates were: June, 73.7% (28/38); July, 63.2% (24/38); August, 64.5% (20/31); September, 72.2% (26/36); October, 73.7% (28/38); November, 65.8% (25/38); January, 68.4% (26/38); and March 43.8% (14/32) (**Table 3**). No significant differences in the temporal prevalence of VTEC were observed (*p* ≥ 0.05).

**Table 3:** Temporal VTEC prevalence rates in heifers.

	Number samples	Detection rate	95% CI^2^	Isolation rate	95% CI^2^
**Overall**					
2012–2015	368	74.7%	69.9–79.0%	70.4%	65.4–74.9%
**Annual**					
2012–2013	103	79.6%	70.3–86.7%	71.8%	62.0–80.0%
2013–2014	111	71.2%	61.7–79.2%	68.5%	58.9–76.8%
2014–2015	154	74.0%	66.2–80.6%	70.8%	62.8–77.7%
**Seasonal**					
Spring	74	79.7%	68.5–87.9%	68.9%	57.0–78.9%
Summer	107	71.0%	61.3–79.2%	67.3%	57.5–75.9%
Fall	112	72.3%	62.9–80.2%	70.5%	61.1–78.6%
Winter	75	78.7%	67.4–87.0%	76.0%	64.5–84.8%
**Monthly^1^**					
June	38	76.3%	59.4–88.0%	73.7%	56.6–86.0%
July	38	63.2%	46.0–77.7%	63.2%	46.0–77.7%
Aug	31	74.2%	55.1–87.5%	64.5%	45.4–80.2%
Sept	36	77.8%	60.4–89.3%	72.2%	54.6–85.2%
Oct	38	73.7%	56.6–86.0%	73.7%	56.6–86.0%
Nov	38	65.8%	48.6–79.9%	65.8%	48.6–79.9%
Jan	38	73.7%	56.6–86.0%	68.4%	51.2–82.0%
Mar	32	65.6%	46.8–80.8%	43.8%	26.8–62.1%


*Spring: Mar-May, Summer: June-Aug, Fall: Sept-Nov, Winter: Dec-Feb. ^*1*^Excludes months not sampled for all three cohort-years (Apr, May, Dec, Feb). ^*2*^95% confidence intervals using the Wilson procedure with a correction for continuity (http://vassarstats.net/prop1.html).*

#### Cohort-Specific

The average per-animal isolation rate was 70.5% [95% CI, 64.2 to 76.8%] – 72.1, 68.8, and 70.8% for years 1, 2, and 3, respectively, with rates ranging from 33.3 to 100.0%. The prevalence of “persistent shedders” as defined by Menrath et al. (2010), i.e., at least half of the samples and ≥4 consecutive sampling being *vt* positive, was 90.0% (9/10), 50.0% (6/12), 62.5% (10/16) for cohorts 1, 2, and 3, respectively or 65.8% (25/38) [95% CI, 48.6 to 79.9%] of total animals sampled. However, isolation rates were not statistically different between individuals classified as “persistent shedders” and “non-persistent shedders” (*p* ≥ 0.05) (**Figure 1** and **Supplementary Table 4**).

Single-event sampling of month-old calves in 2013, 2014, and 2015 yielded overall detection and isolation rates of 78.6% (22/28) [95% CI, 58.5 to 91.0%] and 71.4% (20/28) [95% CI, 51.1 to 86.1%], respectively (**Table 4**).

**Table 4:** Summary of VTEC sampling in calves (single-event).

	April 29′13	May 7′14	May 6′15	Total
Number of animals	7	6	15	28
Number of samples	7	6	15	28
Detection rate	7 (100.0%)	4 (66.7%)	11 (73.3%)	22 (78.6%)
Isolation rate	7 (100.0%)	2 (33.3%)	11 (73.3%)	20 (71.4%)
Number of isolates	28	8	43	79
Number of serotypes	3	3	4	6
Serotypes	O26:NM (6)^∗∗^	O91:H21 (1)	O2:H29 (4)^∗∗^	O111:NM (9)^∗∗^
(Number Of samples)	O6:H34 (1)	O111:NM (1)^∗∗^	O6:H34 (1)	O26:NM (6)^∗∗^
	O130:H38 (1)	O130:H38 (1)	O91:H21 (1)^∗∗^	O2:H9 (4)^∗∗^
			O111:NM (8)^∗∗^	O6:H34 (2)
				O91:H21 (2)^∗∗^
				O130:H38 (2)


*^∗∗^’Clinically significant serotypes (Seropathotype A, B, C). Virulence profiles: O111:NM (*vt1 eae hlyA*), O26:NM (*vt1 eae hlyA*), O2:H29 (*vt2*), O6:H34 (*vt2*), O130:H38 (*vt1 vt2 hlyA saa*), and O91:H21 (*vt1 vt2 hlyA saa*).*

### VTEC Diversity and Persistence

#### Serotype Distribution

Thirty-two VTEC serotypes were isolated among heifers overall – 17, 19, and 14 in each sampling year, respectively. Among 259 VTEC positive samples, the most commonly isolated serotypes were O139:H19 (37.1%, 96), O22:H8 (15.8%, 41), O?:H8 (15.4%, 40), O130:H38 (12.4%, 32), O6:H34 (10.0%, 26), O91:H21 (8.1%, 21), O113:H21 (6.2%, 16), and O28ac:H25 (4.2%, 11). With the exception of O130:H38 and O113:H21, these serotypes were isolated in all three cohort-years. *In silico* serotyping of 14/40 O?:H8 isolates identified all as serotype O108:H8. Serotypes O139:H19, O22:H8 and O?(O108):H8 were among the five most prevalent serotypes in all three years. From a combination of 31 sampling events, they were also the most persistently isolated [O139:H19 (96.8%, 30 months), O?(O108):H8 (77.4%, 24 months) and O22:H8 (74.2%, 23 months)] (**Table 1**). Between years, significant differences (*p* < 0.05) were observed within serotypes O139:H19 (years 1 vs. 3), O130:H38 (years 1 and 2 vs. 3), O6:H34 (years 1 vs. 2 and 3), O91:H21 (years 1 and 2 vs. 3), and O113:H21 (years 2 vs. 3). Differences were also observed within years; the most significant being the predominance of serotype O139:H19 in years 2 and 3. Overall trends included the divergent paths of O139:H19 and O130:H38, where O139:H19 prevalence increased as the latter declined. While not statistically significant (*p* ≥ 0.05), the incidence of other prevalent serotypes also contributed to the variability observed in serotype distribution each year, specifically the decline of O6:H34 and the increased prevalence of O91:H21 and O113:H21 (**Figure 2**).

During the spring, summer, fall and winter months, 17, 21, 18, and 13 serotypes were identified, respectively (**Supplementary Table 5**). While inter-seasonal differences in prevalence were not significant within each serotype (*p* ≥ 0.05), elevated prevalence was observed for serotype O139:H19 in the spring while reduced prevalence was observed in the spring for O22:H8. Within each season, serotype O139:H19 was significantly more prevalent than several of the top serotypes, particularly in the spring (*p* < 0.05) (**Figure 2**).

An average of 4.3 serotypes were isolated per individual animal – 5.9 serotypes (range 4–9), 4.0 serotypes (range 2–6), and 3.6 (range 2–6) serotypes in years 1, 2, and 3, respectively (**Supplementary Table 6**). For 9/10, 10/12, and 16/16 heifers in years 1, 2, and 3, respectively, at least 50% of isolates belonged to the most prevalent serotypes (defined as those isolated from ≥5 samples per year) (**Figure 1** and **Supplementary Table 6**). Within each cohort, no individual animal contributed overwhelmingly more to the prevalence of any serotype (*p* ≥ 0.05). In year 3, serotype O139:H19 was isolated more frequently from heifer 21315, than from 5 others (*p* < 0.05) (**Supplementary Table 6**).

Six serotypes were isolated from calves overall; 6/7 (85.7%) and 8/11 (72.7%) VTEC-containing samples were positive for serotype O26:H11 and O111:NM in 2013 and 2015, respectively (**Table 4**). In contrast, these serotypes were only isolated from 1.2% (3/259) and 0.8% (2/259) of VTEC positive heifers samples, respectively (**Table 1**).

#### Clermont Phylotype Distribution

**FIGURE 3:**
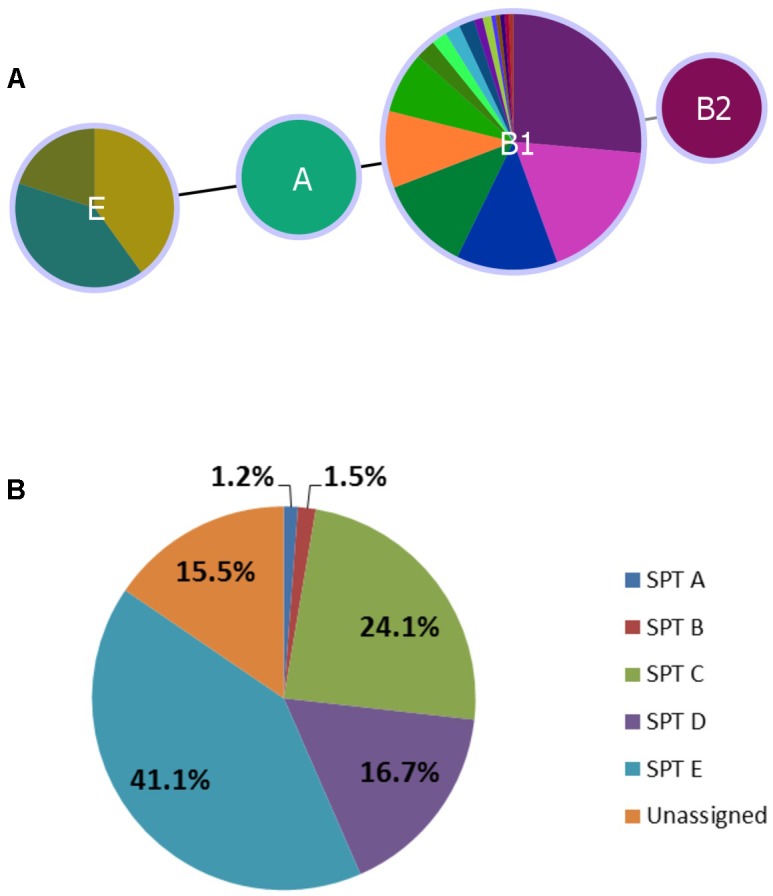
Distribution of Clermont phylotype **(A)** and seropathotype **(B)** among heifer isolates. **(A)** (*n* = 179 strains). Phyloviz2 goeBURST full MST based on presence/absence of *arpA*, *chuA*, *yjaA*, and *TspEF.C2* genes (≥85% sequence identity, ≥60% query length). Phylotype A (*n* = 2, O136:H16), B2 (*n* = 1, O2:H6), E (*n* = 10, O132:H18, O157:H7, O137:H5, O137:H41) and B1 (*n* = 166, remaining serotypes). **(B)** (*n* = 336 strains). Frequency of seropathotypes (SPT) as defined by Karmali et al. (2003): Unassigned: not previously identified as VTEC or not fully typed (*n* = 52); SPT A: high incidence, common in outbreaks, associated with HC and HUS (*n* = 4); SPT B: moderate incidence, uncommon in outbreaks, associated with HC and HUS (*n* = 5); SPT C: low incidence, rare in outbreaks, associated with HC and HUS (*n* = 81); SPT D: low incidence, rare in outbreaks, not associated with HC and HUS (*n* = 56); SPT E: non-human only, not implicated in outbreaks, not associated with HC and HUS (*n* = 138).

*In silico* assessment of 179 isolates spanning all serotypes in the study yielded four phylotypes – A (O136:H16), B2 (O2:H6), E (O132:H18, O157:H7, O137:H5, O137:H41), and B1 (remaining serotypes). Classification was largely serotype-specific, and 92.7% (166/179) of isolates were classified as phylotype B1 (**Figure 3A**). The single isolate from serotype O2:H6 was more specifically classified as subgroup B2_3_ (*chuA*+, *yjaA*+, and *TspE4.C2*+), previously only found in human samples (Carlos et al., 2010).

#### Simpson’s Index of Diversity

The overall serotype diversity, estimated using SID was 0.87–0.88, 0.86, and 0.81 for years 1, 2, and 3, respectively (**Table 1**) and 0.84, 0.90, 0.87, 0.86 for spring, summer, fall and winter, respectively (**Supplementary Table 5**). The average per-animal SID was 0.72 [95% CI, 0.67 to 0.77] – 0.84, 0.73, 0.63 for cohorts 1, 2, and 3, respectively (**Figure 1**).

#### Genomic Diversity

**FIGURE 4:**
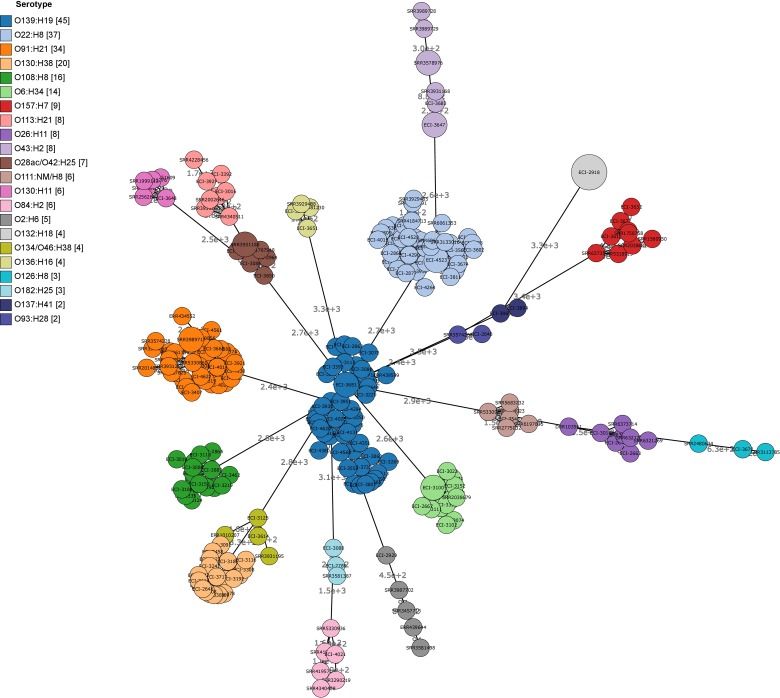
Minimum spanning tree (MST) of wgMLST profiles of heifer isolates and reference strains. (*n* = 251 strains; 179 from this study and 72 SRA references). MST construction and visualization using GrapeTree MSTree2 method using allele calls from Bionumerics *E. coli* wgMLST scheme (8162/17380 loci assessed). Node labels indicate the number of allele differences and bubble size indicates the prevalence of allelic profiles.

**FIGURE 5:**
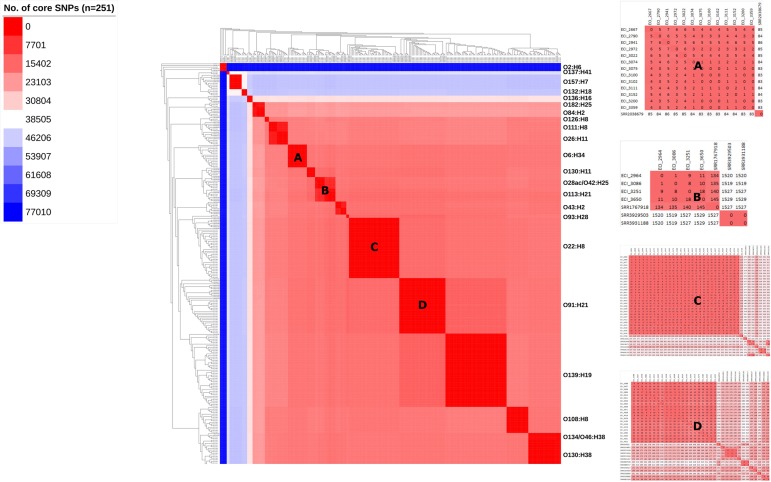
Maximum-likelihood phylogeny and similarity matrix of heifer isolates and reference strains based on pangenome-derived core SNPs. (*n* = 251 strains; 179 from this study and 72 SRA references). SNP discovery using Panseq (‘fragmentationSize’ = ‘500’, ‘percentIdentityCutoff’ = ’85,’ ‘coreGenomeThreshold’ = ‘251,’ ‘runMode’ = ‘pan,’ ‘cdhit’ = ‘1’). Tree construction using PhyML (substitution model selection criterion: AIC, type of tree improvement: NNI, branch support: aLRT SH-like). Tree and heat map visualization using iTOL. Legend: gradient of the no. of core SNPs (applies to main heat map only). Panels: serotype-specific heat map of the similarity matrix between herd and reference strains; **(A)** O6:H34, **(B)** O28ac/O42:H25, **(C)** O22:H8, **(D)** O91:H21.

Cluster analysis by wgMLST and pangenome-derived core SNP analysis delineated strains at the phylotype and serotype level, including epidemiologically unrelated reference strains (**Figures 4**, **5**). Within several major serotype clusters, including O6:H34, O28ac/O42:H25, O22:H8 and O91:H21, reference strains could be further distinguished from the herd isolates based on the number of SNP differences (**Figure 5**). Core SNP analysis identified 161335 SNPs from the pan-genome derived from all 251 strains, including 179 from the herd and 72 NCBI reference strains (**Figure 5**).

### Human Health Risk

#### Seropathotype Distribution

All five seropathotypes (SPT) as defined by Karmali et al. (2003) were identified in this study: SPT A (1.2%, 4/336), SPT B (1.5%, 5/336), SPT C (24.1%, 81/336), SPT D (16.7%, 56/336), SPT E (41.1%, 138/336) (**Figure 3B**). The most commonly identified seropathotype was SPT E, although the majority of these isolates belonged to two serotypes: O139:H19 (69.6%, 96/138) and O130:H38 (23.2%, 32/138) Clinically associated SPT A, B and C serotypes included: A (O157:H7), B (O26:H11, O111:NM), and C (O22:H8, O91:H21, O113:H21, O137:H41, O2:H6) (**Table 5**).

**Table 5:** Seropathotype and serotype distribution of heifer and calf VTEC isolates.

SPT^1^	Definition (Karmali et al., 2003)	Seropathotype reference^2^	Serotype	Number of heifer isolates^3^	Number of calf isolates^3^
A	High incidence in humans, outbreak (common) and HUS/HC associated	Karmali et al., 2003	O157:H7^∗,^^stx1,stx2a^	4	
B	Moderate incidence in humans, outbreak (uncommon) and HUS/HC associated	Karmali et al., 2003	O26:NM(H11)^∗,stx1^	3	6
B		Karmali et al., 2003	O111:NM^∗,stx1^	2	9
C	Low incidence in humans, outbreak (rare) and HUS/HC associated	WHO, 1998	O22:H8^stx1,stx2c^	41	
C		Karmali et al., 2003	O91:H21^stx1,stx2a,c,d^	21	2
C		Karmali et al., 2003	O113:H21^stx2a,c,d^	16	
C		WHO, 1998	O137:H41^stx1,stx2c^	2	
C		WHO, 1998	O2:H6^stx1,stx2^ ^(non-a,cd)^	1	
D	Low incidence in humans, outbreak (rare) associated, not HUS/HC associated	Thompson et al., 2005; Canada	O6:H34^stx2c,d^	26	2
D		Karama et al., 2008	O28ac:H25^stx2a,c,d^	11	
D		Karmali et al., 2003	O132:NM(H18)^stx2a^	8	
D		EFSA, 2013	O130:H11^stx1,stx2a^	4	
D		EFSA, 2013	O43:H2^stx1,stx2a^	2	
D		WHO, 1998	O84:H2^∗stx1^	2	
D		EFSA, 2013	O182:H25^∗stx2a^	2	
D		WHO, 1998	O126:H8^stx1^	1	
E	Non-human only, not outbreak or HUS/HC associated	Karmali et al., 2003	O113:NM^stx2a,c^	1	
E		Sandhu et al., 1996; Renter et al., 2007; cattle, Canada	O139:H19^stx1,stx2a,c,d^	96	
E		Hornitzky et al., 2002; Brett et al., 2003; cattle, Australia	O130:H38^stx1,stx2a,d^	32	2
E		Karmali et al., 2003	O46:H38^stx1,stx2a,c,d^	5	
E		Midgley et al., 1999; Makino et al., 2000; seagulls, Japan; cattle, Australia	O136:H16^stx1^	2	
E		Miyao et al., 1998; cattle, Japan	O42:H25^stx2a,c,d^	2	
UA			O93:H28^stx2a,c,d^	2	
UA			O43:H6^stx1,stx2a^	2	
UA			O152:H38^stx1,stx2a,d^	1	
UA			O130:H12^stx1,stx2a,d^	1	
UA			O137:H5^stx1^	1	
UA			O?(O108):H8^stx1,stx2a,c,d^	40	
UA			OR:H8^stx1,stx2c^	2	
UA			O139:H?^stx1,stx2a,d^	1	
UA			O130:H?^stx1,stx2c,d^	1	
UA			OR:H21^stx2a,c^	1	
C^∗^			O2:H29^†,stx2(nosubtyping)^		4
ALL			ALL	336	25


*UA – unassigned, not previously reported as VTEC or not fully typed (O or H untypeable). (O/H) typing supported by WGS data. ^*1*^Seropathotype (SPT) classification of pathogenicity based on Karmali et al., 2003 scheme. ^*2*^Literature reference supporting seropathotype assignment, e.g., HUS associated (SPT C), human-associated but no HUS/HC (SPT D) (source, country of isolation if applicable and available). ^*3*^336/948 heifer isolates with unique fecal sample-serotype-virulotype combinations; 25/79 calf isolates with unique fecal sample-serotype-virulotype combinations. ^†^Serotype found in calf isolates only. Virulence factors (heifers isolates only): ^∗^*eae* positive, ^*stx1*^*stx1* positive (no subtyping), ^stx2a,stx2c,stx2d^stx2-subtype positive.*

#### Virulence-Associated Genes

##### vt1, vt2, hlyA, eae, saa

**FIGURE 6:**
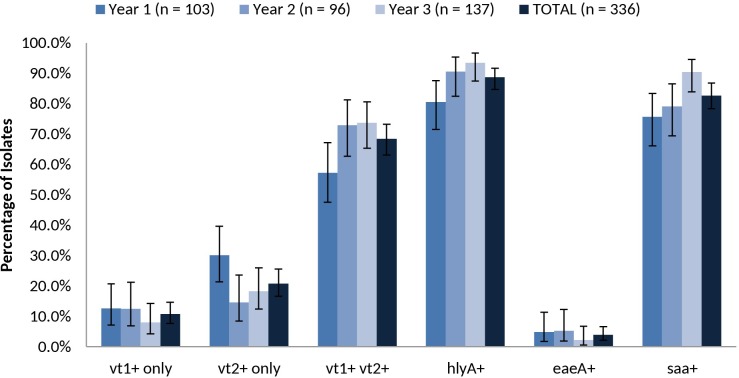
Prevalence of *vt1*, *vt2*, *vt1 vt2*, *hlyA*, *eae*, and *saa* among heifer isolates. (*n* = 336 strains). Error bars representing 95% confidence intervals for proportions were calculated using the Wilson procedure with a correction for continuity (http://vassarstats.net/prop1.html).

The overall prevalence of virulence-associated genes *vt1* only, *vt2* only, *vt1 vt2*, *hlyA, eae* and *saa* were 10.7% (36/336), 20.8% (70/336), 68.5% (230/336), 88.7% (298/336), 3.9% (13/336), and 82.7% (278/336), respectively (**Figure 6**). Relative carriage rates were consistent in all years, with most isolates carrying *vt1 vt2*, *hlyA* and *saa*. The *eae* gene was present in 100.0% (*n* = 9) of SPTs A and B isolates (O157:H7, O26:H11, O111:NM) but absent from 100.0% of SPT C (*n* = 81) isolates (O22:H8, O91:H21, O113:H21, O137:H41, O2:H6) and 92.9% (52/56) of SPT D isolates (e.g., O6:H34, O28ac:H25, O132:H18, O130:H11) (**Supplementary Table 8**). Carriage of the virulence-associated genes was largely serotype-specific. For example, 99.0% (95/96) of serotype O139:H19 isolates were *vt1*+ *vt2*+ *eae-hlyA*+ *saa+* and 100% (26/26) of serotype O6:H34 isolates were *vt1-vt2*+ *eae- hlyA-saa-*. The exception was serotype O22:H8, where 58.5% (24/41) were *vt1*+ *vt2-eae*- *hlyA*+ *saa+* and 41.5% (17/41) also carried *vt2*; the latter profile was isolated almost exclusively in year 3 (**Supplementary Table 7**).

##### *In silico* analysis of additional virulence factors

**FIGURE 7:**
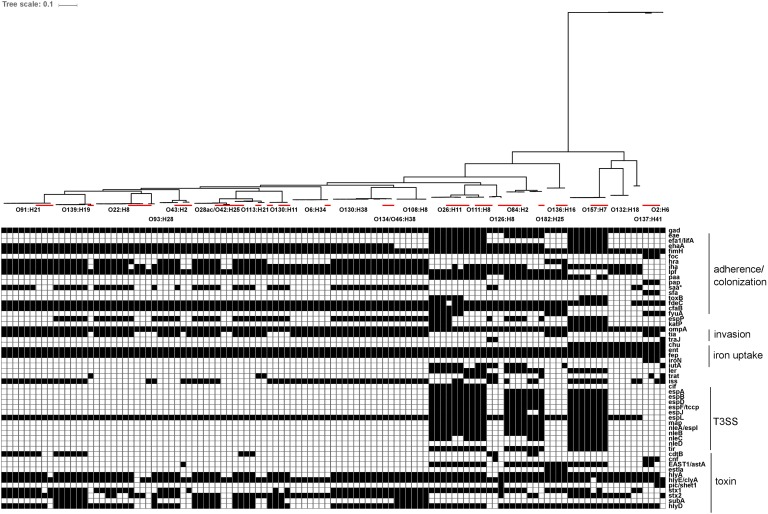
*In silico* presence/absence of virulence-associated genes vs. core SNP derived maximum-likelihood phylogeny of heifer isolates. (*n* = 115 strains). Maximum-likelihood phylogeny from Panseq core SNPs (‘fragmentationSize’ = ‘ 500,’ ‘percentIdentityCutoff = ’85,’ ‘coreGenomeThreshold’ = 115, ‘runMode’ = ‘pan,’ ‘cdhit’ = ‘1’) generated using PhyML (substitution model selection criterion: AIC, type of tree improvement: NNI, branch support: aLRT SH-like). Binary presence/absence called with Panseq (query file https://github.com/phac-nml/ecoli_vf/blob/master/data/repaired_ecoli_vfs.ffn, ‘percentIdentityCutoff’ = ’85,’ ‘coreGenomeThreshold’ = ‘251’) using 2710 virulence-associated gene sequences, including gene variants. Only genes present in at least one isolate were included. Gene presence/absence was confirmed in ABRicate v0.7 using the VFDB database (2597 sequences), with the following parameters (% coverage ≥60, % identity ≥80). Red line: reference strains from the NCBI SRA. *gad(X)* (transcriptional regulator), *eae* (intimin), *efa1/lifA* (EHEC factor for adherence/lymphocyte inhibitory factor), *ehaA* (autotransporter), *fimH* (type 1 fimbrin adhesin), *foc(A-D, F-H,Y)* (F1C fimbriae), *hra* (heat-resistant agglutinin), *iha* (adhesin), *lpf* (long polar fimbriae), *paa* (porcine attaching effacing associated protein), *pap(A-K)* (P fimbriae), *saa* (STEC autoagglutinating adhesin), *sfa(A-H, S, X, Y)* (S fimbriae), *toxB* (toxin B), *fdeC* (factor adherence *E. coli*), *cfaB* (colonization factor; CFA/I fimbrial subunit B), *fyuA* (pesticin receptor), *espP* (serine protease), *katP* (catalase-peroxidase), *ompA* (outer membrane protein A), *tia* (invasion determinant), *traJ* (conjugal transfer protein), *chu(A, S-Y)* (*E. coli* hemin uptake), *ent(A-F, S)* (enterobactin), *fep(A-E, G)* (enterobactin), *iroN* (siderophore receptor), *iutA* (ferric aerobactin receptor), *ler* (LEE encoded regulator), *trat* (complement resistance and surface exclusion outer membrane protein), *iss* (increased serum survival), *cif* (T3SS effector; cycle-inhibiting factor), *espA* (T3SS effector), *espB* (T3SS effector), *espD* (T3SS effector), *espF/tccp* (T3SS effector), *espJ* (T3SS effector), *espL* (T3SS effector), *map* (T3SS effector; mitochondria-associated protein), *nleA/espI* (non-LEE T3SS effector), *nleB* (non-LEE T3SS effector), *nleC* (non-LEE T3SS effector), *nleD* (non-LEE T3SS effector), *tir* (T3SS effector; translocated intimin receptor), *cdtB* (cytolethal distending toxin B), *cnf* (cytoxic necrotizing factor 1), EAST1*/astA* (ST; heat stable enterotoxin 1), *estIa* (ST; heat stable enterotoxin 1), *hlyA* (hemolysin), *hlyE/clyA* (hemolysin E), *pic/shET1* (set1A, set1B) (serine protease autotransporter/Shigella enterotoxin), *stx1* (Shiga toxin 1), *stx2* (Shiga toxin 2), *subA* (subtilase cytotoxin), *hlyD* (hemolysin secretion protein D).

Genetic sequences for previously described virulence factors with roles in adherence/colonization, invasion, iron uptake, type III secretion systems and toxin production were identified in all serotypes recovered in this study. Carriage of virulence factors was largely serotype-specific within the strains isolated in this study, relative to reference strains. Of interest, strains from human illness-associated serotypes (O157:H7, O26:H11 and O111:NM) shared similar virulence profiles with two serotypes which are not typically associated with human illness (O182:H25, O84:H2). Specifically, genes coding for components found in the bacterial type III secretion system were also found in the latter group. Colonization factor gene *cfab* was found in all strains from all serotypes except O2:H6, O137:H41, O132:H18, and O157:H7. Alternative adherence factor genes *iha* and *lpfA* were found in 88.9% (64/72) and 93.1% (67/72) of herd strains, respectively (**Figure 7**).

##### *In silico* analysis of *eae* and *hlyA* genetic diversity

**FIGURE 8:**
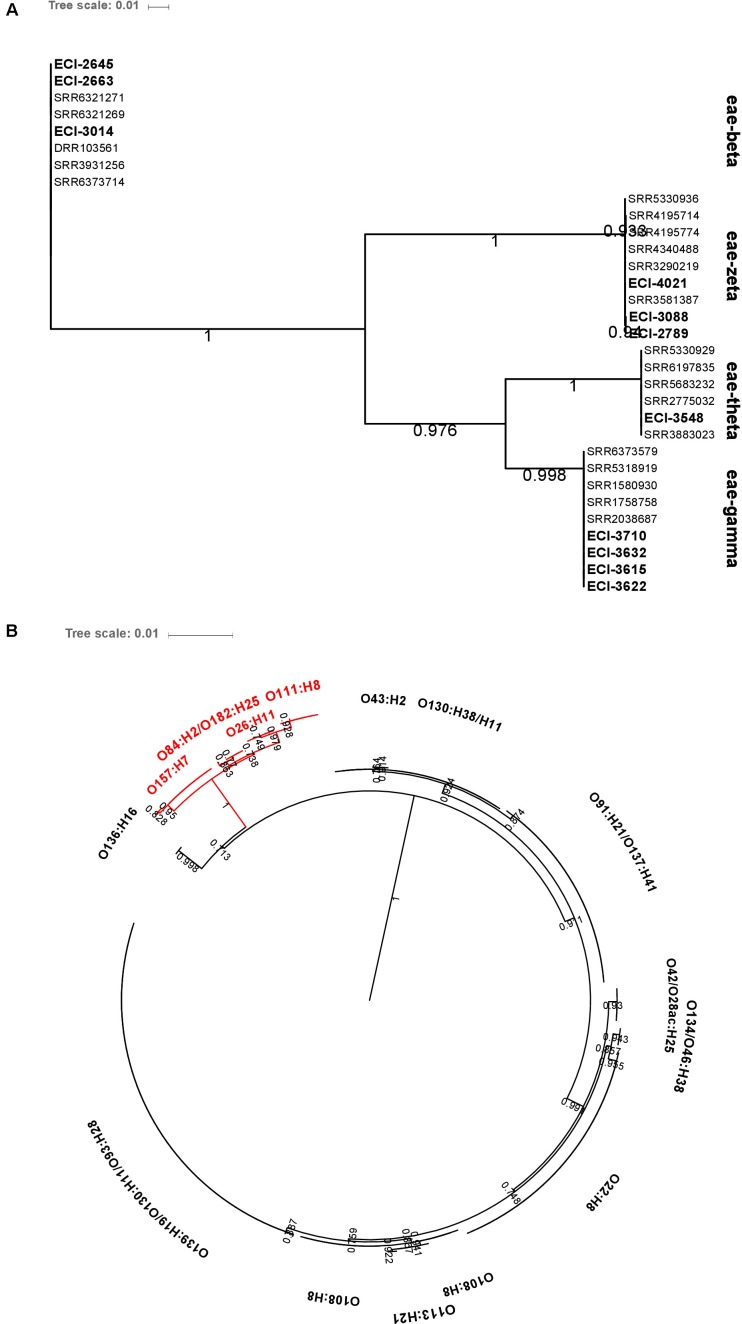
Maximum-likelihood phylogenetic trees of **(A)** *eae* and **(B)** *hlyA* genetic diversity among heifer isolates (*eae*, *n* = 32; *hlyA*, *n* = 190). BLAST parameters (≥ 85% sequence identity, ≥ 60% query length). Alignment by MAFFT. Maximum-likelihood tree construction using phyML (substitution model selection criterion: AIC, type of tree improvement: NNI, branch support: aLRT SH-like). aLRT SH-like branch support values are indicated on each node. Tree visualization using iTOL. **(B)** Red text: *eae*-positive strains.

Intimin-β, ζ, θ, and γ were identified in O26:H11, O84:H2 and O182:H25, O111:H8, and O157:H7 isolates, respectively. The genetic diversity of *hlyA* was largely serotype-specific with some serotypes (O139:H19 and O93:H28; O130:H38 and O130:H11) harboring highly related variants. Particularly, intimin-positive isolates (O157:H7, O84:H2, O182:H25, O26:H11, and O111:H8) shared related *hlyA* variants which clustered separately from those identified in all other serotypes (**Figure 8**).

##### *vt2* subtypes

**FIGURE 9:**
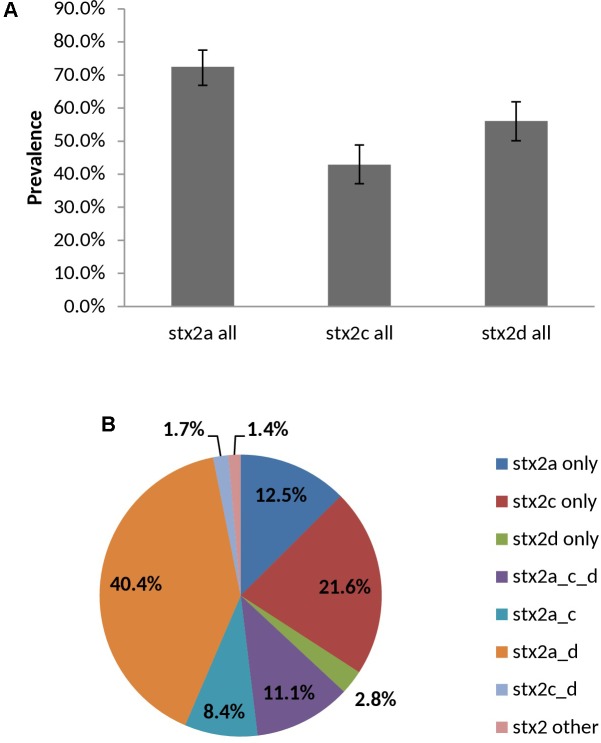
Prevalence **(A)** and distribution **(B)** of *vt2* subtypes among heifer isolates. (*n* = 287 strains). **(A)** Error bars representing 95% confidence intervals for proportions were calculated using the Wilson procedure with a correction for continuity (http://vassarstats.net/prop1.html). *stx2* other: PCR negative for *stx2a*, *c*, and *d* variants.

98.6% (283/287) of *vt2*-positive isolates were experimentally typed as *vt2a*, *vt2c* and/or *vt2d* by PCR – subtypes which have been associated with severe illness. Overall prevalence rates for *vt2a*, *vt2c*, *vt2d* were 72.5, 42.9, and 56.1%, respectively, with 61.7% (177/287) of isolates being positive for multiple subtypes (**Figure 9**). Subtyping of *vt2* was largely serotype-specific; for example, 91.2% (83/91) of O139:H19 *vt2* positive isolates carried *vt2a* and *vt2d* and 94.1% (16/17) of O22:H8 *vt2* positive isolates carried subtype *vt2c* (**Supplementary Table 7**).

#### *In silico* Analysis of Antibiotic Resistance (AR) Genes

**FIGURE 10:**
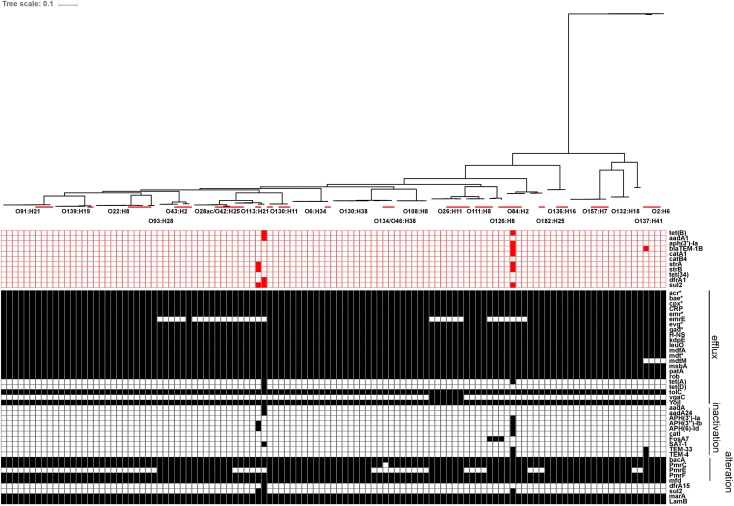
*In silico* presence/absence of antibiotic resistance (AR) genes vs. core SNP derived maximum-likelihood phylogeny of heifer isolates. (*n* = 115 strains). Maximum-likelihood phylogeny from Panseq core SNPs (‘fragmentationSize’ = ‘ 500,’ ‘percentIdentityCutoff = ‘85,’ ‘coreGenomeThreshold’ = 115, ‘runMode’ = ‘pan,’ ‘cdhit’ = ‘1’) generated using PhyML (substitution model selection criterion: AIC, type of tree improvement: NNI, branch support: aLRT SH-like). AR gene detection: ABRicate v0.7 (% coverage 60, %id 80) ResFinder (2228 sequences, red) and CARD (2153 sequences, black) databases. Red line: reference strains from the NCBI SRA. **ResFinder:** *tet*(B) (tetracycline efflux), *aadA1* (aminoglycoside nucleotidyltransferase), *aph(3′)-Ia* (aminoglycoside phosphotransferase), *blaTEM-1B* (Beta-lactamase), *catA1* (chloramphenicol acetyltransferase), *catB4* (chloramphenicol acetyltransferase), *strA* (aminoglycoside phosphotransferase), *strB* (aminoglycoside phosphotransferase), *tet*(34) (tetracycline resistance), *dfrA1* (dihydrofolate reductase), *sul2* (sulfonamide resistant dihydropteroate synthase). **CARD:** *acr(A,B,D,E,F,S)* (antibiotic efflux; multiple antibiotic resistance), *bae(R,S)* (regulates *pmr* polymyxin resistance), *cpx(A,R)* (regulatory; antibiotic efflux; multiple antibiotic resistance), CRP (global regulator; antibiotic efflux; multiple antibiotic resistance), *emr(A,B,D,K,R,Y)* (antibiotic efflux; multiple antibiotic resistance), *emrE* (antibiotic efflux; multidrug transporter), *evg(A,S)* (regulatory; antibiotic efflux; multiple antibiotic resistance), *gad(W,X)* (regulatory; antibiotic efflux; multiple antibiotic resistance), H-NS (global gene regular; multiple antibiotic resistance), *kdpE* (transcriptional activator; regulatory; antibiotic efflux), *leuO* (transcription factor; antibiotic efflux), *mdfA* (multidrug efflux pump), *mdt(A,B,C,E,F,G,H,L,N,O,P)* (multidrug efflux complexes), *mdtM* (antibiotic efflux; multiple antibiotic resistance), *msbA* (multidrug resistance transporter), *patA* (ATP-binding cassette transporter; fluoroquinolone resistance), *rob* (positive regular for acrAB efflux genes; multiple antibiotic resistance), *tet*(A) (tetracycline efflux pump), *tet*(D) (tetracycline efflux pump), *tolC* (multidrug efflux complex subunit), *vgaC* (streptogramin resistance), *yojI* (peptide antibiotic microcin J25 resistance), *aadA* (aminoglycoside nucleotidyltransferase), *aadA24* (aminoglycoside nucleotidyltransferase), *APH(3′)-Ia* (*aphA-1*; aminoglycoside phosphotransferase), *APH(3″)-Ib* (*strA*, *orfH*; aminoglycoside phosphotransferase), *APH(6)-Id* (*strB*, *orfI*; aminoglycoside phosphotransferase), *catI* (chloramphenicol acetyltransferase), *FosA7* (fosfomycin thiol transferase), *SAT-1* (streptothricin-acetyltransferase), *TEM-33* (*IRT-*5; inhibitor-resistant beta-lactamase), *TEM-4* (extended-spectrum beta-lactamase), *bacA* (bacitracin resistance), *pmrC* (polymyxin resistance), *pmrE* (*ugd*; polymyxin resistance), *pmrF* (*arnC*; polymyxin resistance), *mfd* (transcription-repair coupling factor; fluoroquinolone resistance), *dfrA15* (diaminopyrimidine resistance), *sul2* (sulfonamide resistant dihydropteroate synthase), *marA* (global activator protein; multiple antibiotic resistance), *lamB* (porin; negative regular for AR; multiple antibiotic resistance).

Using the ResFinder database, genes for acquired antibiotic resistance were detected in 1/72 representative herd strains (1.4%); specifically one O113:H21 strain (ECI-3927) carried genes associated with resistance to tetracycline [*tet*(B)], streptomycin/spectinomycin (*aadA1*), trimethoprim (*dfrA1*) and sulfonamide (*sul2*). Additional screening using the CARD database yielded hits for 65 genes associated with resistance to several drug classes; the majority of these being involved in antibiotic efflux. Of these genes, 48/65 were found in at least 95% of strains, including epidemiologically unrelated reference strains, 6/65 were only found in reference strains, and 7/65 genes were only found in one specific O113:H21 strain (ECI-3927), the majority of which corresponded to the AR genes also identified by ResFinder in the same strain. The remaining 3/65 genes corresponded to *emrE* present in 57/72 strains, *vgaC* in 3/72 strains, and *prmE* in 37/72 strains. All three of these genes were serotype-specific: *emrE*, associated with multidrug-resistance, was detected in all strains from serotypes O108:H8, O111:H8, O130:H11, O130:H38, O132:H18, O134/O46:H38, O136:H16, O137:H41, O139:H19, O157:H7, O182:H25, O2:H6, O22:H8, O6:H34, O91:H21, and O93:H28; all three strains encoding *vgaC*, associated with streptogramin and pleuromutilin resistance, were serotype O26:H11; and *prmE*, associated resistance to peptide antibiotics, was detected in all strains from serotypes O126:H8, O130:H11, O130:H38, O132:H18, O136:H16, O157:H7, O2:H6, O26:H11, O28ac/O42:H25, O43:H2, O6:H34, and O84:H2. The serotype specificity of these genes also extended to the reference strains (**Figure 10**).

#### Calves

Human disease-associated serotypes O26:H11, O111:NM and O91:H21 were isolated from calf samples, with O26:NM and O111:NM being present in 85.7% (6/7) and 72.7% (8/11) of VTEC positive calves in 2013 and 2015, respectively (**Table 4**). Virulence profiles were strictly serotype-specific: O111:NM (9/9) *vt1 eae hlyA*, O26:NM (6/6) *vt1 eae, hlyA*, O2:H29 (4/4) *vt2*, O6:H34 (2/2) *vt2*, O130:H38 (2/2) *vt1 vt2 hlyA saa*, and O91:H21 (2/2) *vt1 vt2 hlyA saa*.

## Discussion

Despite a general recognition of cattle as an important reservoir for VTEC, there are still knowledge gaps pertaining to the ecology of the organism, including their prevalence, distribution and persistence in a herd, as well as that of particular serotypes and virulence factors. In this study, we tried to address some of these questions through year-long surveillance of each of three consecutive cohorts of cattle within a closed herd.

### VT-Immunoblot Method for Isolation of VTEC From Unenriched Cattle Fecal Samples

The VT-immunoblot method (VT-IB) has been successfully used for the isolation of VTEC from enrichment cultures of ground beef (Atalla et al., 2000), surface water samples (Johnson et al., 2014; Nadya et al., 2016; Falardeau et al., 2017) and enriched cattle feces (Karama et al., 2008). This study represents the first use for unenriched cattle feces. Used in conjunction with the VT-ELISA, recovery rates were relatively high (i.e., 94.2% of samples with a positive VT-immunoblot signal yielded a VTEC isolate) in contrast to methods which yielded high detection but low isolation rates for VTEC (Pradel et al., 2000; Jenkins et al., 2002; Renter et al., 2007; Bosilevac and Koohmaraie, 2011; Monaghan et al., 2011; Paddock et al., 2012). A similar study investigating the long term prevalence of VTEC in cattle using *stxA*-specific colony hybridization found that *stx*-positive colonies were isolated from 60.5% of PCR positive pre-screening cultures (Geue et al., 2002). In this study, we have demonstrated for the majority of samples, that pre-enrichment was neither necessary for isolate recovery nor for capturing the serotype diversity within samples. In fact, VTEC was more likely to be isolated from positive samples using the direct method. The elimination of enrichment reduces the turnaround time from sample to isolate by at least 6–24 h and may help prevent the enrichment culture bias observed during the isolation of VTEC, which is in agreement with other studies involving the isolation of pathogenic *E. coli* (Conrad et al., 2016), *Listeria monocytogenes* and *Salmonella* spp (Gorski, 2012).

### VTEC Prevalence

A range of non-O157 VTEC prevalence rates in beef cattle has been reported worldwide, from 4.0 to 71.7% in pastures, feedlots, and at slaughter (Pradel et al., 2000; Schurman et al., 2000; Hussein, 2007; Baltasar et al., 2014; Mir et al., 2015). In Canada, similar studies surveying all VTEC from individual cattle have reported rates of 4.0 to 24.7% (Wilson et al., 1992; Schurman et al., 2000; Karama et al., 2008). Additionally, while closed herds have previously been sampled for O157:H7 (Gannon et al., 2002) and other top 7 VTEC serogroups (Hallewell et al., 2016), to our knowledge, this is the first longitudinal study of overall VTEC prevalence in a closed herd in Canada. A comparable study in Germany surveyed the long term prevalence of VTEC on four farms managed as closed herds over three years and found that VTEC prevalence varied widely between herds – from 29 to 82% (Geue et al., 2002). However, comparisons of prevalence should be made with caution due to the variability in target population and methodology, particularly between molecular detection and isolation-based studies. The relatively high rate of VTEC isolated in this study (70.4%) may be attributed to the age of the sampled cohort, as prevalence has been shown to be higher in calves and heifers (Wilson et al., 1992; Cobbold and Desmarchelier, 2000; Orden et al., 2002), as well as the greater sensitivity of the methods used. In contrast, Mir et al. (2015) found a higher prevalence of VTEC in cows than heifers while Blanco et al. (1997) observed no age effect on VTEC prevalence.

The absence of temporal peaks in the overall prevalence of non-O157 VTEC has been previously reported in cattle (Ennis et al., 2012). Conversely, seasonality was observed by Jenkins et al. (2002), where prevalence was highest in July and lowest in September and by Monaghan et al. (2011) who observed a surge in late summer-early autumn. However, only about 5 and 53.4% of the detected samples had yielded a confirmed isolate in each study, respectively. Seasonal variations in prevalence have been observed in “top 7” human disease-associated *E. coli* serogroups (Pearce et al., 2006; Stanford et al., 2016), such as O26, O45, O103, O111, O121, O145, and O157. However, prevalence was either evaluated by PCR detection only or isolates were not VTEC in general. Compared to VTEC O157:H7, for which the seasonal link between increased prevalence in cattle and disease incidence during the summer and fall months is generally established (Van Donkersgoed et al., 1999; Tarr et al., 2005; Blanco et al., 2008; CDC, 2012), more research is warranted to investigate temporal trends of non-O157 VTEC. Cohort-specific VTEC prevalence was variable in all years, with some individuals being persistent VTEC-shedders and others only shedding sporadically. Recent studies have defined “super shedders” as not just individuals shedding at least 10^4^ cfu per gram of feces as per the original definition (Chase-Topping et al., 2008) but also those that shed persistently over a period of time (Lim et al., 2007; Carlson et al., 2009; Menrath et al., 2010). Specifically, Lim et al. (2007) and Carlson et al. (2009) defined this as shedding for more than three consecutive months and Menrath et al. (2010) as having at least half of the samples and ≥4 consecutive sampling being *stx* positive. According to the latter definition and in the absence of direct quantification of the level of shedding, almost two-thirds of the individuals in this study could be classified as a “super shedder.” Indeed, the phenomenon of super or persistent shedding in non-O157 VTEC has been reported elsewhere (Menrath et al., 2010; Murphy et al., 2016). However, statistically significant differences in the frequency of VTEC shedding were not observed between any two individuals. This may be due to the small sample sizes, which may have been ameliorated with more frequent sampling. Additionally, the “non-super shedders” were generally within 1–2 positive samples of satisfying these requirements, demonstrating the potential pitfalls of categorically assigning individuals to groups using criteria derived from studies which may differ in experimental methods and design. Most notably, in Menrath et al. (2010), VTEC prevalence was based on the PCR detection of *stx* in fecal samples, whereas it was based on the isolation of VTEC in this study. Nevertheless, the regular detection of VTEC in clinically healthy cattle herds is well known (Geue et al., 2002).

### VTEC Diversity and Serotype Persistence

VTEC diversity was assessed by cluster analysis using multiple methods with varying levels of resolution – specifically, serotyping, phylotyping, wgMLST and pangenome-derived core SNP analysis. From 38 heifers, 948 isolates or 336 unique strains (i.e., those with unique sample-serotype-virulotypes profiles) were classified into 32 serotypes, yielding high serotype diversity (SID 0.87). In a similar study involving longitudinal sampling of a single herd, 45 different serotypes were identified from only 94 strains (Jenkins et al., 2002). However, in that study, more than 700 samples were collected from random sampling of an extensive grazing beef cattle farm with a high turnover rate. Similarly, a 20-week study of a large abattoir turning over more than 1,000 cattle a day recovered 24 serotypes from 27 VTEC isolates (Karama et al., 2008). In contrast, Bettelheim et al. (2005) sampled a cohort of 30 steers on two separate occasions to obtain a total of 474 *E. coli* isolates, classified into 52 serotypes. This suggests that repeat sampling of individual animals within a closely related herd with a low turnover rate may contribute to decreased serotype diversity, particularly as the rumen microbiota stabilizes as the animal matures (Jami et al., 2013). The predominance of phylotype B1 in this study is consistent with findings that suggest it is prevalent in ruminants (Carlos et al., 2010), particularly among cattle isolates (Coura et al., 2015; Askari Badouei et al., 2016).

Genomic diversity assessed at the whole genome level, using both wgMLST and SNP methods, showed clear delineation at the serotype level, although herd strains from this study were distinguishable from epidemiologically unrelated reference strains of the same serotype, based on the number of allele and SNP differences. In most cases, virulence factor and antimicrobial gene profiles were also conserved within each serotype, among the herd strains. This may suggest that each serotype is actually represented by a limited number of clones which have been transmitted both within and between cohorts. Serotype-specific cluster analyses may elucidate any phylogenetic substructures within each serotype, which could suggest an evolution of these strains within groups of cattle over time.

Serotypes O139:H19, O22:H8 and O?(O108):H8 were persistently isolated throughout the study. *E. coli* O22:H8 has previously been isolated from ground beef and chicken products (Mora et al., 2007; Alonso et al., 2016; Cadona et al., 2016), domestic cats (Bentancor et al., 2007), cattle (Blanco et al., 2004; Farah et al., 2007; Gonzalez et al., 2016) and HUS patients (Blanco et al., 2003) and was also repeatedly isolated in another study of non-O157 VTEC in cattle (Menrath et al., 2010). A recent study by Martorelli et al. (2017) showed that *E. coli* O22:H8 is able to interfere with *E. coli* O157:H7 *in vitro* and *in vivo* based on their superior growth rates, biofilm-forming abilities and adherence properties and may reduce the overall susceptibility of calves to O157:H7 colonization and shedding. The presence of this organism could potentially explain the low percentage of *E. coli* O157:H7 isolations from this herd. Further investigation of the association between *E. coli* O22:H8 and *E. coli* O157:H7 in cattle herds is clearly warranted. *E. coli* O139 is typically associated with animals and mild disease in humans (Sandhu et al., 1996; Wang et al., 2005; EFSA, 2013). However, Toro et al. (2015) showed that an insertion sequence (IS) excision enhancer (IEE) shown to promote genomic rearrangements and strain diversification in EHEC O157:H7, was identical in sequence to the IEE found in O139:H38/NM strains. In the former study, the simultaneous presence of the IEE and IS629 was proposed to be a marker for strains with enhanced pathogenic potential.

In the 1950s, Sears et al. (1950, 1956) described “resident” *E. coli* serotypes that were observed over several months in an individual but that were vulnerable to replacement by new resident isolates over time in humans and dogs. In our study, the potential “residency” of certain serotypes was reflected not just between cohort-years but also at the individual animal level; in all but 3/38 cows surveyed, the most prevalent serotypes in each year, including O139:H19, O22:H8 and O?(O108:H8), contributed at least 50% of the VTEC isolates for each animal. Apparent population turnover was also observed. Serotype O130:H38 was highly prevalent in the first two years but absent in year 3. Conversely, serotype O91:H21 was rarely isolated early in the study but became a dominant serotype in the last year. Temporal variability and persistence of serotypes within a herd has been described previously (Tokhi et al., 1993; Beutin et al., 1997; Jenkins et al., 2002), but to our knowledge, this is the first study showing that non-O157 VTEC serotypes can persist through multiple cohorts within a distinct population of cattle. Geue et al. (2002) monitored the long term prevalence of VTEC in five groups of cattle among four farms from birth to slaughter, over three years; in their study specific VTEC clones of serotypes O26:H11 and O165:H25 were found to have persisted in herds. Taken together, this may suggest that persistence of serotypes or clones may occur in the same cohorts over multiple years (Geue et al., 2002) or across multiple cohorts, as in the current study.

### Human Health Risk

#### Seropathotypes

Seropathotype classifications, based on reported occurrences of outbreak and sporadic illness and incidence of HUS (Karmali et al., 2003), showed that while SPT A and SPT B VTEC were isolated in this study, the majority of isolates were of SPT D, E or otherwise unassigned. This may suggest that there is a low risk to humans from most VTEC isolated from these cattle. However, since non-O157 VTEC are not routinely isolated in many public health labs, it may also reflect a knowledge gap in our understanding of the contribution of other VTEC serotypes to human illness. The remainder, accounting for almost 25% of isolates, belonged to five serotypes classified as SPT C, which have been shown to be associated with HUS and may represent emerging pathogens.

#### Virulence Factors

Virulence profiles consisting of *vt1*, *vt2*, *hlyA*, *eae*, and *saa* were largely serotype-specific, suggesting the circulation of a single dominant clone within serotypes (Beutin et al., 1997; Jenkins et al., 2002). The most common *vt* type was *vt1 vt2*, comprising 68.5% (230/336) of isolates, which has been previously reported as the top *vt* type in surveys of dairy cattle (Irino et al., 2005; Gonzalez et al., 2016). In contrast, Mir et al. (2015) found 70.0% of all STEC encoded *vt2* only, with *vt1 vt2* being the least common genotype. Predominant *vt* types have been shown to vary between farms (Cobbold and Desmarchelier, 2001), differ between dairy and beef cattle (Cerqueira et al., 1999) and be serotype-specific in distinct cattle populations (Montenegro et al., 1990; Beutin et al., 1997); indeed, multiple *vt* types were only observed in serotype O22:H8. The *vt2* subtypes *a*, *c* and/or *d* were identified in 98.6% of *vt2* positive isolates – subtypes which have been associated with the development of haemorrhagic colitis or HUS (Orth et al., 2007; Kawano et al., 2008), as well as increased toxin potency *in vitro* and *in vivo* (Fuller et al., 2011). High levels of these subtypes in bovine isolates have been previously reported in Australia (Brett et al., 2003) and the US (Shridhar et al., 2017), and may represent a pool of transmissible virulence factors that could increase the pathogenicity of strains that acquire them. Additional studies should also be conducted to determine whether toxin-negative strain populations of the VTEC serotypes identified in this study co-exist with their toxin-positive counterparts; these could represent a reservoir of potential human pathogens, or alternatively could indicate that certain sub-populations are not likely to be associated with human disease.

In this study, 88.7% of VTEC carried the EHEC haemolysin gene (EHEC-*hlyA*), which is also known to be highly prevalent in human clinical VTEC (Beutin et al., 2004). A survey of cattle herds in Iran detected EHEC-*hlyA* in 11.9% (*n* = 452) of VTEC isolates (Askari Badouei et al., 2016) and in a longitudinal study of closed cattle herds in Germany, the prevalence of EHEC-*hlyA* was 26.5% (*n* = 1,647) (Geue et al., 2002). In our study, genetic diversity was also observed within the EHEC-*hlyA* gene itself. Notably, serotypes with known pathogenic potential and encoding *eae*, shared a variant that clustered distinctly from other serotype-specific variants. This supports previous findings where RFLP patterns for the EHEC-haemolysin gene were generally serotype-specific and also delineated by the presence/absence of *eae* (Boerlin et al., 1998; Askari Badouei et al., 2016). The identification of a number of other virulence-associated genes with roles in adherence, colonization, invasion, iron uptake and toxin production in the majority of strains suggests the potential to cause human disease exists amongst many of these commensal cattle isolates.

Intimin is an outer membrane adhesin encoded by the *eae* gene on the pathogenesis island called LEE (locus for enterocyte effacement). It mediates intimate attachment with host cells (McDaniel and Kaper, 1997) and is strongly associated with known pathogenic serotypes and severe disease in humans (Donnenberg et al., 1993; Boerlin et al., 1999; Blanco et al., 2004). Carriage of *eae* was observed in only 3.8% of isolates in this study and supports previous reports of low carriage of intimin in bovine isolates (Blanco et al., 1997; Geue et al., 2002; Irino et al., 2005; Farah et al., 2007; Karama et al., 2008; Menrath et al., 2010; Ennis et al., 2012) as well as the minor role played by intimin in the colonization of the bovine host by most VTEC (Ramachandran et al., 2003). Intimin may be essential for causing severe human illness by certain strains but may be replaced by alternatives, such as the *saa*-encoded STEC autoagglutinating adhesin, in other VTEC (de Azavedo et al., 1994; Paton et al., 1996; Paton et al., 2001). In this study, *eae* was absent from 100.0% of SPT C and 95.0% of SPT D isolates which are associated with sporadic but severe illness in humans and minor diarrheal illness, respectively. This further suggests alternative modes of colonization, including putative genetic determinants for host cell adherence *iha* (Tarr et al., 2000) and *lpfA* (Doughty et al., 2002), which were detected in 88.9% (64/72) and 93.1% (67/72) of isolates screened with the extended virulence factor panel in this study, respectively. Recent studies have shown that *saa* and *eae* may be mutually exclusive in single VTEC strains (Aidar-Ugrinovich et al., 2007; Farah et al., 2007; Ennis et al., 2012; Lee et al., 2017). In this study, *saa* was present in 82.7% of isolates (none of which also encoded *eae*), including several SPT C serotypes previously associated with severe human illness: O22:H8, O91:H21, O113:H21, O137:H41 and O2:H6. Similarly, all 13 intimin-positive isolates (O157:H7, O26:11 and O111:NM, O182:H25, O84:H2) were negative for *saa*.

#### Pathotypes

Diarrheagenic *E. coli* (DEC) are classified into six pathotypes based on differences in disease manifestation, mode of infection and the presence of certain hallmark virulence factors: enterotoxigenic *E. coli* (ETEC), enteroinvasive *E. coli* (EIEC), enteropathogenic *E. coli* (EPEC), enterohemorrhagic *E. coli* (EHEC), enteroaggregative *E. coli* (EAEC), and diffusely adherent *E. coli* (DAEC). In this study, EHEC (encoding LEE genes, *vt*, *hlyA*) of serotypes O157:H7, O26:H11, O111:H8, O182:H25, and O84:H2 were identified. Serotypes O157:H7, O26:H11, and O111:H8 are part of the “top 7” priority serotypes most frequently implicated in severe illness (Karmali et al., 2003), while O182:H25 and O84:H2 are emerging EHEC that have been previously isolated from humans (Friedrich et al., 2002; Cookson et al., 2006; Brandt et al., 2011; Delannoy et al., 2013). Interestingly, all serotypes excluding O2:H6, O137:H41, O132:H18, and O157:H7 carried the *cfaB* gene, which codes for part of the colonization factor CFA/I; colonization factors are important virulence determinants which mediate ETEC adherence. However, genes encoding heat-stable toxins (STs) characteristic of ETEC strains (e.g., *estIa*), were only found in serotype O136:H16. One strain of serotype O2:H6 was phylogenetically distinct from all other strains and encoded genes *pic/shet1*, characteristic of EAEC; however, genes for aggregative adherence fimbriae (AAF), were absent. The isolation of VTEC that carry virulence factors typically attributed to different pathotypes suggests either emerging pathogens or the possible exchange of virulence factors between groups of potentially pathogenic *E. coli* (Beutin et al., 1997; Reid et al., 2000; Wick et al., 2005; Bettelheim and Goldwater, 2014). Indeed, VTEC/ETEC hybrids have been previously detected in humans and cattle (Nyholm et al., 2015), and perhaps the most well-known example of an emerging pathogenic hybrid is the O104:H4 European outbreak of 2011, where an enteroaggregative *E. coli* O104:H4 strain acquired the *Stx*-2 bacteriophage and caused over 4,000 illnesses, and 50 deaths (Grad et al., 2012). Cattle EHEC-*hlyA* encoding strains, which constituted the majority of strains in our study, may be a potential source of DEC strains of intermediate pathotypes (Askari Badouei et al., 2016).

#### Antibiotic Resistance

Owing to the dominance of lab-based antimicrobial susceptibility testing (van Belkum and Dunne, 2013) and relative novelty of whole-genome based predictions of antimicrobial resistance, it is difficult to assess the distribution of antibiotic resistance genes in this study in the context of similar studies, although high concordance between phenotypic and predicted susceptibility has been demonstrated in several bacterial species (Stoesser et al., 2013; Zankari et al., 2013; Tyson et al., 2015; Neuert et al., 2018). The detection of acquired antibiotic resistance genes in one strain (O113:H21, SPT C) in this study [tetracycline, *tet*(B); streptomycin/spectinomycin, *aadA1*; trimethoprim, *dfrA1* and sulfonamide, *sul2*], suggests that cattle may contribute multidrug resistant VTEC of clinically associated serotypes albeit at low levels.

### VTEC in Calves

Minimal interpretation of the calf VTEC data can be made as sampling occurred only once per year. However, the prevalence of VTEC falls within the wide range of rates previously observed in calves; from 20 to 95.6% (Tokhi et al., 1993; Shaw et al., 2004; Baltasar et al., 2014). Overall detection and isolation rates (78.6 and 71.4%) were similar to those observed in yearling heifers, which supports the findings of Baltasar et al. (2014) but are contrary to those of Mir et al. (2016), who found that VTEC colonization decreased with age and that this was correlated with lower gut microflora diversity in younger animals. Low serotype diversity and a high prevalence of SPT B isolates, specifically O26:NM and O111:NM were observed in calves. These serotypes have previously been associated with severe illness and occasional outbreaks (Karmali et al., 2003) and were rarely isolated from the heifers in this study. Previous studies have also reported a low concordance between dam and calf serotype populations (Pearce et al., 2004; Shaw et al., 2004), suggesting that the calf microbiome differs from that of the yearlings (Jami et al., 2013; Mir et al., 2016), and may allow different VTEC types to flourish. The early introduction and high prevalence of serotype O26 in calves have also been reported in Scotland and New Zealand (Pearce et al., 2004; Shaw et al., 2004; Jaros et al., 2016), with a possible decline in frequency as the animals age (Shaw et al., 2004). Baltasar et al. (2014) suggested that particular VTEC strains may have a propensity for the colonization of immature gastrointestinal systems. Direct sampling of dam-calf pairs would be required to elucidate the transmission dynamics of VTEC in calves.

## Conclusion

In summary, we have demonstrated the efficacy of the VT-immunoblot method for the recovery of VTEC from unenriched cattle feces; VTEC was successfully recovered from 94.2% of samples with a positive immunoblot signal. VTEC prevalence and serotype diversity were relatively high among the yearling heifers and calves within this closed herd and patterns of multi-year persistence were observed for certain serotypes, particularly O139:H19, O?(O108):H8, and O22:H8. The majority of cattle could be described as “persistent VTEC shedders,” although significant differences in VTEC prevalence were not observed among animals. Statistically significant temporal trends were observed among several prevalent serotypes but not for overall VTEC prevalence. Intra-cohort contributions of individuals to serotype prevalence were not significantly different. The majority of isolates were classified as phylotype B1 and although all seropathotypes were identified, the majority of isolates belonged to SPT C (24.0%) or E (40.9%). Higher risk pathogenic serotypes known to cause illness did not dominate in the yearling heifers but based on limited sampling, may be prevalent in calves, with implications for cohort-specific mitigation efforts for VTEC transfer. Carriage of virulence factors *vt1*, *vt2*, *eae*, *hlyA*, and *saa* was largely serotype-specific; the majority of strains carried *vt1 vt2 hlyA* and *saa*, while *eae* was restricted to recognized pathogenic serotypes O157:H7, O26:H11, and O111:NM but also O84:H2 and O182:H25. Elevated prevalence of EHEC-*hlyA* in the predominately non-EHEC strain collection and the carriage of ETEC specific virulence factors supports the potential for intermediate or hybrid pathotypes and/or the exchange of virulence factors between potentially pathogenic *E. coli*. *In silico* assessment of an extended panel of virulence factors identified the presence of genes associated with adherence, colonization, invasion, iron uptake and toxin production. Acquired antibiotic resistance genes identified by ResFinder were observed in one strain belonging to a serotype of clinical significance (O113:H21). High levels of clonality within serotypes at the whole genome level, combined with the serotype-specificity of virulence and AR gene content, suggests the presence of single dominant clones that are transmitted within and among cohorts. This may indicate the existence of common farm-level sources of contamination that persist across years, that some VTEC occupy certain niches, and that the introduction of new members to distinct cattle populations is limited. Importantly, while strains generally clustered phylogenetically by serotype, differences at the gene and SNP level within each serotype enables the examination of a distinct VTEC population at two levels beyond serotype. We have demonstrated the utility of multi-year cohort sampling for investigating the extent of VTEC and serotype persistence and generated a relatively unique collection of isolates to support future analyses of the rate of genomic change over time, among different serotypes. Future work should also focus on the genetic factors which promote intra-herd persistence and exclusion of particular serotypes and the applicability of these results to the broader ecology of VTEC in cattle.

## Author Contributions

RJ developed the original VT-immunoblot assays used for screening environmental and food samples for VTEC and provided training and advice on experimental procedures and necessary reagents. KZ’s laboratory carried out wet lab serotyping and toxin typing of all VTEC isolates and provided reference strains. CJ adapted the original VT-immunoblot methods for the Lethbridge laboratory, designed and established the sampling strategy and oversaw all aspects of the study prior to June 2013. LYRW and CJ contributed to the collection of samples, isolation of VTEC, and curation of the strain collection. LYRW contributed to the whole genome sequencing. LYRW and CL performed the analyses. LYRW wrote the first draft of the manuscript. All authors contributed to the conception and design of the study, manuscript revision, and read and approved the submitted version.

## Conflict of Interest Statement

The authors declare that the research was conducted in the absence of any commercial or financial relationships that could be construed as a potential conflict of interest.

## Footnotes

1 https://github.com/tseemann/shovill

2 https://github.com/lh3/seqtk

3 https://github.com/chadlaing/Panseq

4 http://www.atgc-montpellier.fr/phyml/

5 http://itol.embl.de/

6 https://github.com/phac-nml/ecoli_vf/blob/master/data/repaired_ecoli_vfs.ffn

7 https://github.com/tseemann/abricate

8 https://mafft.cbrc.jp/alignment/server/

9 http://vassarstats.net/prop1.html

## Abbreviations

AR: antibiotic resistance
BCIG: 5-bromo-4-chloro-3-indolyl-ß-D-glucuronide
BCIP: 5-bromo-4-chloro-3-indolyl-phosphate, immunoblot substrate
DAEC: diffusely adherent *E. coli;* DEC, diarrheagenic *E. coli*
ELISA: enzyme-linked immunosorbent assay
EAEC: enteroaggregative *E. coli;* EHEC, enterohaemorrhagic *E. coli;* EIEC, enteroinvasive *E. coli*
EPEC: enteropathogenic *E. coli;* ETEC, enterotoxigenic *E. coli*
HC: haemorrhagic colitis
HGMF: hydrophobic grid-membrane filter
HRP: horseradish peroxidase, enzyme conjugate for ELISA
HUS: haemolytic uremic syndrome
LEE: locus of enterocyte effacement
(wg)MLST: (whole genome) multilocus sequence typing
mTSB/A-VC: modified tryptic soy broth/agar
TSB/A supplemented with bile salts: vancomycin and cefsulodin
NBT: nitroblue tetrazolium chloride, immunoblot substrate
PBS: phosphate buffer saline
PCR: polymerase chain reaction
SID: Simpson’s Index of Diversity
SNP: single nucleotide polymorphism
SPT: seropathotype
ST: heat-stable toxin
TMB: tetramethylbenzidine, ELISA substrate
V(S)TEC/STEC: verotoxigenic (shigatoxigenic) *Escherichia coli*
WGS: whole genome sequencing
