# Supplementary material for: Multi-Year Persistence of Verotoxigenic Escherichia coli (VTEC) in a Closed Canadian Beef Herd: A Cohort Study
Source: Front Microbiol. 2018 Aug 31;9:2040. doi: 10.3389/fmicb.2018.02040 (PMC6127291; doi:10.3389/fmicb.2018.02040)
Supplement: Supplementary file 1 [file Table_1.docx]

| Supplementary Table 1. Primers used in this study. | | | | | |  |  | |  |
| --- | --- | --- | --- | --- | --- | --- | --- | --- | --- |
|  |  | |  |  | |  |  | |  |
| **Gene** | **Primer Name** | | **Specificity** | **Gene location** | | **Sequence (5'-3')** | **Amplicon Size (bp)** | | **Reference** |
|  | | | | | | | | | |
| *stx1/vt1* | vt1-F | conserved region of *vt1* | | 639–659 | CATTGTCTGGTGACAGTAGCT | | 732 | Gannon et al, 1997 | |
|  | vt1-R |  | | 1371–1351 | CCCGTAATTTGCGCACTGAG | |  |  |  |
|  | stx1F | subunit coding region of *stx1* | | 454–633 | ATAAATCGCCATTCGTTGACTAC | | 180 | Paton and Paton, 1998 | |
|  | stx1R |  | |  | AGAACGCCCACTGAGATCATC | |  |  |  |
| *stx2/vt2* | vt2-F | conserved region of *stx2* | | 624–644 | CCATGACAACGGACAGCAGTT | | 779 | Gannon et al, 1997 | |
|  | vt2-R |  | | 1403–1384 | CCTGTCAACTGAGCACTTTG | |  |  |  |
|  | stx2F | subunit coding region of *stx2* | | 603–857 | GGCACTGTCTGAAACTGCTCC | | 255 | Paton and Paton, 1998 | |
|  | stx2R | (including variants) | |  | TCGCCAGTTATCTGACATTCTG | |  |  |  |
| *eaeA* | eaeAF | conserved between EPEC | | 27–410 | GACCCGGCACAAGCATAAGC | | 384 | Paton and Paton, 1998 | |
|  | eaeAR | and VTEC | |  | CCACCTGCAGCAACAAGAGG | |  |  |  |
| *hlyA* | hlyAF | EHEC hlyA | | 70–603 | GCATCATCAAGCGTACGTTCC | | 534 |  |  |
|  | hlyAR |  | |  | AATGAGCCAAGCTGGTTAAGCT | |  |  |  |
| *saa* | SAADF | *saa* | |  | CGTGATGAACAGGCTATTGC | | 119 | Paton et al, 2001 | |
|  | SAADR |  | |  | ATGGACATGCCTGTGGCAAC | |  |  |  |
| *stx2a/vt2a* | stx2a-F2 | *stx2/vt2* subtype *a* | | 754–774 | GCGATACTGRGBACTGTGGCC | |  | Scheutz *et al*., 2012 | |
|  | stx2a-R3 |  | | 1,079–1,102 | CCGKCAACCTTCACTGTAAATGTG | | 349 |  |  |
|  | stx2a-R2 |  | | 1,079–1,100 | GCCACCTTCACTGTGAATGTG | | 347 |  |  |
| *stx2c/vt2c* | stx2c-F1 | *stx2/vt2* subtype c | | 926–955 | GAAAGTCACAGTTTTTATATACAACGGGTA | | 177 |  |  |
|  | stx2c-R2 |  | | 1,079–1,102 | CCGGCCACYTTTACTGTGAATGTA | |  |  |  |
| *stx2d/vt2d* | stx2d-F1 | *stx2/vt2* subtype d | | 927-955 | AAARTCACAGTCTTTATATACAACGGGTG | |  |  |  |
|  | stx2d-R1 |  | | 1085-1105 | TTYCCGGCCACTTTTACTGTG | | 179 |  |  |
|  | stx2d-O55R |  | | 1140-1161 | TCAACCGAGCACTTTGCAGTAG | | 235 |  |  |
|  | stx2d-R2 |  | | 1184-1206 | GCCTGATGCACAGGTACTGGAC | | 280 |  |  |
