# Supplementary material for: Multi-Year Persistence of Verotoxigenic Escherichia coli (VTEC) in a Closed Canadian Beef Herd: A Cohort Study
Source: Front Microbiol. 2018 Aug 31;9:2040. doi: 10.3389/fmicb.2018.02040 (PMC6127291; doi:10.3389/fmicb.2018.02040)
Supplement: Supplementary file 2 [file Table_2.DOCX]

| Supplementary Table 2 . Number of serotypes from unenriched vs. enriched VT-immunoblot method comparison (n = 64 samples). | | | |
| --- | --- | --- | --- |
|  |  |  |  |
| **Unenriched only** | **Enriched only** | **Both methods** |  |
| **7** | **4** | **11** |  |
| O93:H28 | O88:H25 | O130:H38 |  |
| O2:H6 | O130:H8 | O6:H34 |  |
| O26:NM | OR:H8 | O139:H19 |  |
| O152:H38 | O130:H34 | O?:H8 |  |
| O130:H25 |  | O182:H25 |  |
| O139:H? |  | O132:NM |  |
| O130:H12 |  | O22:H8 |  |
|  |  | O113:H21 |  |
|  |  | O28ac:H25 |  |
|  |  | O46:H38 |  |
|  |  | O91:H21 |  |
