# Supplementary material for: Multi-Year Persistence of Verotoxigenic Escherichia coli (VTEC) in a Closed Canadian Beef Herd: A Cohort Study
Source: Front Microbiol. 2018 Aug 31;9:2040. doi: 10.3389/fmicb.2018.02040 (PMC6127291; doi:10.3389/fmicb.2018.02040)
Supplement: Supplementary file 3 [file Table_3.DOCX]

| Supplementary Table 3. Serotype diversity of samples selected for in-depth sampling by unenriched vs. enriched VT-immunoblot (n = 10 samples). | | | | | |
| --- | --- | --- | --- | --- | --- |
|  |  |  |  |  |  |
| **Sample^1^** | **Total No. isolates** | **No. of isolates - Unenriched** | **Serotype(s) - Unenriched** | **No. of isolates - Enriched** | **Serotype(s) - Enriched** |
| Faecal-1469 | 19 | 11 | O6:H34 | 8 | O6:H34 |
|  |  |  | **O?:H8** |  |  |
| Faecal-1474 | 22 | 11 | O22:H8 | 11 | O22:H8 |
|  |  |  | O6:H34 |  | O6:H34 |
| Faecal-1478 | 17 | 7 | O139:H19 | 10 | O139:H19 |
|  |  |  |  |  | **O28ac:H25** |
| Faecal-1480 | 22 | 10 | O139:H19 | 12 | O139:H19 |
|  |  |  | **O28ac:H25** |  |  |
| Faecal-1485 | 26 | 15 | O139:H19 | 11 | O139:H19 |
| Faecal-1486 | 24 | 13 | O130:H38 | 11 | O130:H38 |
| Faecal-1487 | 29 | 12 | O130:H38 | 17 | O130:H38 |
|  |  |  | O?:H8 |  | O?:H8 |
| Faecal-1488 | 25 | 13 | O6:H34 | 12 | O6:H34 |
|  |  |  | O22:H8 |  | O22:H8 |
| Faecal-1492 | 14 | 6 | O91:H21 | 8 | O91:H21 |
|  |  |  | O130:H38 |  | O130:H38 |
|  |  |  | O113:H21 |  | O113:H21 |
|  |  |  | O139:H19 |  | O139:H19 |
| Faecal-1493 | 49 | 27 | O91:H21 | 24 | O91:H21 |
|  |  |  |  |  |  |
| ^1^ Samples from which ≥ 5 colonies per method were tested | | | |  |  |
| **Bolded** - Serotype recovered from one method and not the other | | | |  |  |
