# Supplementary material for: Multi-Year Persistence of Verotoxigenic Escherichia coli (VTEC) in a Closed Canadian Beef Herd: A Cohort Study
Source: Front Microbiol. 2018 Aug 31;9:2040. doi: 10.3389/fmicb.2018.02040 (PMC6127291; doi:10.3389/fmicb.2018.02040)
Supplement: Supplementary file 4 [file Table_4.DOCX]

| Supplementary Table 4. VTEC isolation status of individual heifers on each sampling date. | | | | | | | | | | | | | | | | | | | | | | | | |  | |  |  |  |  |  |  |  |  |  |
| --- | --- | --- | --- | --- | --- | --- | --- | --- | --- | --- | --- | --- | --- | --- | --- | --- | --- | --- | --- | --- | --- | --- | --- | --- | --- | --- | --- | --- | --- | --- | --- | --- | --- | --- | --- |
|  |  |  |  |  |  |  |  |  |  |  |  |  |  | |  |  |  |  |  |  |  |  |  |  |  | |  |  |  |  |  |  |  |  |  |
|  | **2012** | | | | | | | | **2013** | | | | | | | | | | | | | **2014** | | | | | | | | | | | | **2015** | |
| **Cow ID** | **04-10/23** | **05-14** | **06-18** | **07-09** | **08-08** | **09-24** | **10-22** | **11-26** | **01-14/28** | **02-11** | **02-25 to 03-27** | **Cow ID** | | **04-9/23** | **06-10** | **07-08** | **08-15** | **09-23** | **10-21** | **11-13** | **12-09** | **01-09** | **03-02 to 03-26** | **Cow ID** | | **05-26** | **06-24** | **07-22** | **08-18** | **09-29** | **10-21** | **11-14** | **12-09** | **01-16** | **02-27 to 03-19** |
| 21131 | 1 | 0 | 1 | 1 | 1 | 1 | 1 | 1 | 1 | 1 | 1 | 21223 | | 1 | 1 | 1 | 1 | 1 | 1 | 1 | 1 | 1 | 0 | 21309 | | 1 | 1 | 1 | N | 1 | 1 | 1 | 1 | 1 | 1 |
| 21146 | N | 1 | 1 | 1 | 1 | 1 | 0 | 1 | 1 | 1 | 1 | 21206 | | 1 | 1 | 1 | 1 | 1 | 1 | 1 | 1 | 1 | N | 21315 | | 1 | 1 | 1 | 1 | 1 | 1 | 1 | 1 | 1 | 1 |
| 21102 | 1 | 1 | 1 | 1 | 1 | 0 | 1 | 1 | 1 | 1 | N | 21239 | | 1 | 1 | 1 | 1 | 1 | 1 | 0 | 1 | 0 | 1 | 21308 | | 1 | 1 | 1 | 1 | 1 | 1 | 1 | 1 | 1 | 0 |
| 21128 | N | 1 | 1 | 1 | N | 1 | 0 | 1 | 1 | 1 | 1 | 21225 | | N | 1 | 1 | 1 | 1 | 1 | 1 | 1 | 0 | 1 | 21331 | | 1 | 0 | 1 | 1 | 1 | 1 | 1 | 1 | 1 | 1 |
| 21101 | 1 | 1 | 0 | 0 | 0 | 1 | 1 | 1 | 1 | 1 | 1 | 21203 | | 1 | 0 | 1 | 1 | N | 1 | 1 | 1 | 1 | N | 21327 | | 1 | 1 | 1 | 0 | 1 | 1 | 1 | 1 | 0 | 1 |
| 21127 | 1 | 0 | 1 | 0 | 1 | 1 | 1 | 1 | 1 | 1 | 0 | 21210 | | 1 | 1 | 1 | 1 | N | 1 | 0 | 1 | 1 | N | 21335 | | 1 | 1 | 1 | 0 | 1 | 1 | 1 | 1 | 1 | 0 |
| 21120 | 1 | 1 | 1 | 0 | 1 | 0 | 1 | 1 | 1 | 1 | N | 21218 | | 1 | 1 | 1 | 0 | 0 | 1 | 0 | 1 | 1 | 0 | 21317 | | 1 | 1 | 1 | N | 1 | 1 | 0 | 1 | 1 | 0 |
| 21141 | N | 1 | 0 | 0 | 0 | 1 | 1 | 1 | 1 | 1 | 0 | 21204 | | 0 | 1 | 1 | 0 | 1 | 1 | 0 | 1 | 1 | 0 | 21333 | | 1 | 1 | 1 | N | 0 | 1 | 1 | 1 | 1 | 0 |
| 21103 | 1 | 0 | 0 | 0 | 0 | 0 | 1 | 1 | 1 | 1 | N | 21226 | | 1 | 1 | 0 | 1 | 0 | 0 | 1 | 0 | 0 | 1 | 21334 | | 1 | 1 | 0 | N | 1 | 1 | 1 | 1 | 1 | 0 |
| 21108 | 0 | 1 | 1 | 0 | 0 | 1 | 1 | 0 | 0 | 1 | 0 | 21251 | | N | 0 | 0 | 1 | 1 | 0 | 0 | 1 | 1 | 0 | 21323 | | 1 | 1 | 1 | 1 | 1 | 0 | 0 | 0 | 1 | 1 |
|  |  |  |  |  |  |  |  |  |  |  |  | 21209 | | 1 | 0 | 1 | 1 | 1 | 0 | 0 | 0 | 0 | 0 | 21329 | | 1 | 0 | 0 | N | 1 | 1 | 1 | 1 | 0 | 0 |
|  |  |  |  |  |  |  |  |  |  |  |  | 21230 | | N | 1 | 1 | 1 | 0 | 0 | 0 | 0 | 0 | N | 21314 | | 1 | 1 | 0 | 0 | 0 | 0 | 0 | 1 | 1 | 1 |
|  |  |  |  |  |  |  |  |  |  |  |  |  | |  |  |  |  |  |  |  |  |  |  | 21318 | | 1 | 1 | 0 | 1 | 1 | 0 | 0 | 1 | 0 | 0 |
|  |  |  |  |  |  |  |  |  |  |  |  |  | |  |  |  |  |  |  |  |  |  |  | 21341 | | 1 | 0 | 1 | 0 | 1 | 1 | 1 | 0 | 0 | 0 |
|  |  |  |  |  |  |  |  |  |  |  |  |  | |  |  |  |  |  |  |  |  |  |  | 21328 | | 1 | 0 | 0 | N | 0 | 1 | 1 | 0 | 1 | 0 |
|  |  |  |  |  |  |  |  |  |  |  |  |  | |  |  |  |  |  |  |  |  |  |  | 21311 | | 1 | 1 | 0 | 0 | 0 | 0 | 0 | 1 | 0 | 1 |
| N = not tested | | | | |  |  |  |  |  |  |  |  | |  |  |  |  |  |  |  |  |  |  |  | |  |  |  |  |  |  |  |  |  |  |
