# Supplementary material for: Multi-Year Persistence of Verotoxigenic Escherichia coli (VTEC) in a Closed Canadian Beef Herd: A Cohort Study
Source: Front Microbiol. 2018 Aug 31;9:2040. doi: 10.3389/fmicb.2018.02040 (PMC6127291; doi:10.3389/fmicb.2018.02040)
Supplement: Supplementary file 5 [file Table_5.DOCX]

| Supplementary Table 5.Temporal distribution of VTEC serotypes (no. of isolates) among heifers. | | | | | | | | | | |
| --- | --- | --- | --- | --- | --- | --- | --- | --- | --- | --- |
|  |  |  |  |  |  |  |  |  |  |  |
| Serotype | Spring | Summer | Fall | Winter | 2012-2013 | 2013-2014 | 2014-2015 | Total |  |  |
| O139:H19 | 27 | 21 | 29 | 19 | 18 | 30 | 48 | 96 |  |  |
| O22:H8** | 3 | 12 | 15 | 11 | 10 | 9 | 22 | 41 |  |  |
| O?(O108):H8^3^ | 10 | 8 | 11 | 11 | 15 | 9 | 16 | 40 |  |  |
| O130:H38 | 5 | 6 | 13 | 8 | 19 | 13 |  | 32 |  |  |
| O6:H34 | 7 | 5 | 8 | 6 | 15 | 4 | 7 | 26 |  |  |
| O91:H21** | 6 | 9 | 4 | 2 | 2 | 1 | 18 | 21 |  |  |
| O113:H21** | 5 | 2 | 4 | 5 | 3 |  | 13 | 16 |  |  |
| O28ac:H25 | 1 | 2 | 6 | 2 | 4 | 4 | 3 | 11 |  |  |
| O132:NM(H18)^4^ |  | 6 | 2 |  | 4 | 4 |  | 8 |  |  |
| O46:H38 | 2 |  | 1 | 2 | 3 | 2 |  | 5 |  |  |
| O130:H11 |  | 1 | 2 | 1 |  | 4 |  | 4 |  |  |
| O157:H7** |  | 3 | 1 |  |  | 4 |  | 4 |  |  |
| O26:NM(H11)** | 2 |  | 1 |  | 3 |  |  | 3 |  |  |
| O42:H25 |  | 1 | 1 |  |  | 2 |  | 2 |  |  |
| O43:H2 |  |  | 2 |  |  | 2 |  | 2 |  |  |
| O43:H6 |  | 1 | 1 |  |  | 2 |  | 2 |  |  |
| O84:H2 |  | 2 |  |  |  |  | 2 | 2 |  |  |
| O93:H28 | 1 | 1 |  |  | 1 |  | 1 | 2 |  |  |
| O111:NM** |  | 2 |  |  |  | 1 | 1 | 2 |  |  |
| O137:H41** | 1 | 1 |  |  |  |  | 2 | 2 |  |  |
| O136:H16 |  | 2 |  |  |  | 2 |  | 2 |  |  |
| O182:H25 | 1 |  | 1 |  | 2 |  |  | 2 |  |  |
| OR:H8 | 1 |  |  | 1 |  |  | 2 | 2 |  |  |
| O2:H6** |  | 1 |  |  | 1 |  |  | 1 |  |  |
| O113:NM |  |  | 1 |  | 1 |  |  | 1 |  |  |
| O126:H8 |  | 1 |  |  |  | 1 |  | 1 |  |  |
| O130:H? |  | 1 |  |  |  | 1 |  | 1 |  |  |
| O130:H12 | 1 |  |  |  | 1 |  |  | 1 |  |  |
| O137:H5 | 1 |  |  |  |  |  | 1 | 1 |  |  |
| O139:H? | 1 |  |  |  |  | 1 |  | 1 |  |  |
| O152:H38 |  |  |  | 1 | 1 |  |  | 1 |  |  |
| OR:H21 |  |  |  | 1 |  |  | 1 | 1 |  |  |
| Total No. Isolates | 75 | 88 | 103 | 70 | 103 | 96 | 137 | 336 |  |  |
| Total No. Serotypes | 17 | 21 | 18 | 13 | 17 | 19 | 14 | 32 |  |  |
| % Pos Samples^1^ | 68.9% | 67.3% | 70.5% | 76.0% | 71.8% | 68.5% | 70.8% | 70.4% |  |  |
| Total No. Pos Samples^1^ | 51 | 72 | 79 | 57 | 74 | 76 | 109 | 259 |  |  |
| Total No. of Samples | 74 | 107 | 112 | 75 | 103 | 111 | 154 | 368 |  |  |
| SID^2^ | 0.84 | 0.90 | 0.87 | 0.86 | 0.88 | 0.86 | 0.81 | 0.87 |  |  |
| Spring: Mar-May, Summer: June-Aug, Fall: Sept-Nov, Winter: Dec-Feb | | | | | | | | | |  |
| ^1^Exluding samples positive by detection only but for which no isolates were recovered | | | | | | |  |  |  |  |
| ^2^ Simpson's Diversity Index=(1-*D*), where D = (∑n(n-1))/(N(N-1)), n = no. of isolates and N = total no. of isolates | | | | | | | | | |  |
| ^3^(n=14) were typed as O108:H8 using whole genome sequencing and *in silico* serotyping | | | | | | | | | |  |
| ^4^(n=4) were typed as O132:NM using whole genome sequencing and *in silico* serotyping | | | | | | | | | |  |
| **'Clinically associated serotypes | | | | | | | | | |  |
