# Supplementary material for: Multi-Year Persistence of Verotoxigenic Escherichia coli (VTEC) in a Closed Canadian Beef Herd: A Cohort Study
Source: Front Microbiol. 2018 Aug 31;9:2040. doi: 10.3389/fmicb.2018.02040 (PMC6127291; doi:10.3389/fmicb.2018.02040)
Supplement: Supplementary file 6 [file Table_6.DOCX]

| Supplementary Table 6. Intra-herd serotype distribution among heifers. | | | | | | | | | | | | | |  |  |  |  |  |  |  |  |  |  |  |  |  |
| --- | --- | --- | --- | --- | --- | --- | --- | --- | --- | --- | --- | --- | --- | --- | --- | --- | --- | --- | --- | --- | --- | --- | --- | --- | --- | --- |
|  |  |  |  |  |  |  |  |  |  |  |  |  |  |  |  |  |  |  |  |  |  |  |  |  |  |  |
|  | **Year 1 Cow ID** | | | | | | | | | |  |  |  | **Year 2 Cow ID** | | | | | | | | | | | |  |
| Serotype | 21101 | 21102 | 21103 | 21108 | 21120 | 21127 | 21128 | 21131 | 21141 | 21146 |  |  | Serotype | 21203 | 21204 | 21206 | 21209 | 21210 | 21218 | 21223 | 21225 | 21226 | 21230 | 21239 | 21251 |  |
| No. isolates | 15 | 11 | 8 | 5 | 12 | 10 | 12 | 12 | 8 | 11 | **SUM** |  | No. isolates | 10 | 8 | 13 | 5 | 12 | 7 | 9 | 10 | 5 | 3 | 10 | 4 | **SUM** |
| **O139:H19** | 1 |  | 2 | 2 | 4 | 5 | 1 | 1 | 1 | 1 | 18 |  | **O139:H19** | 1 | 5 | 2 | 3 | 5 | 1 | 2 | 4 | 3 | 1 | 3 |  | 30 |
| **O22:H8** | 2 |  | 2 | 1 |  |  | 1 |  |  | 4 | 10 |  | **O22:H8** |  |  | 2 |  | 2 |  | 5 |  |  |  |  |  | 9 |
| **O?(O108):H8** | 2 | 4 |  | 1 | 2 | 2 | 1 |  | 2 | 1 | 15 |  | **O?(O108):H8** |  |  |  | 1 | 2 | 5 |  |  |  |  |  | 1 | 9 |
| **O130:H38** | 2 |  | 3 |  | 2 | 1 | 2 | 3 | 4 | 2 | 19 |  | **O130:H38** |  | 1 | 6 |  |  |  | 2 | 1 |  |  |  | 3 | 13 |
| **O6:H34** | 6 | 5 |  |  | 1 |  | 2 | 1 | 1 |  | 16 |  | O6:H34 | 4 |  |  |  |  |  |  |  |  |  |  |  | 4 |
| O91:H21 |  |  |  |  |  |  | 1 |  |  | 1 | 2 |  | O91:H21 |  |  | 1 |  |  |  |  |  |  |  |  |  | 1 |
| O113:H21 | 1 |  |  |  |  |  | 2 |  |  |  | 3 |  | O28ac:H25 |  |  |  |  |  |  |  |  |  |  | 4 |  | 4 |
| O28ac:H25 |  |  | 1 |  |  |  |  | 3 |  |  | 4 |  | O132:NM(H18) | 1 |  | 1 |  |  |  |  |  | 1 | 1 |  |  | 4 |
| O132:NM(H18) |  | 1 |  |  | 1 |  | 1 |  |  | 1 | 4 |  | O46:H38 |  |  |  |  |  | 1 |  |  | 1 |  |  |  | 2 |
| O46:H38 |  |  |  |  | 1 | 1 |  |  |  | 1 | 3 |  | O130:H11 | 3 |  |  |  |  |  |  |  |  |  | 1 |  | 4 |
| O26:NM |  | 1 |  |  | 1 |  | 1 |  |  |  | 3 |  | O157:H7 |  |  |  | 1 | 1 |  |  | 1 |  | 1 |  |  | 4 |
| O93:H28 |  |  |  |  |  | 1 |  |  |  |  | 1 |  | O42:H25 |  |  |  |  |  |  |  |  |  |  | 2 |  | 2 |
| O182:H25 | 1 |  |  |  |  |  |  | 1 |  |  | 2 |  | O43:H2 |  | 1 |  |  |  |  |  | 1 |  |  |  |  | 2 |
| O2:H6 |  |  |  |  |  |  |  | 1 |  |  | 1 |  | O43:H6 |  |  |  |  |  |  |  | 2 |  |  |  |  | 2 |
| O113:NM |  |  |  | 1 |  |  |  |  |  |  | 1 |  | O111:NM |  | 1 |  |  |  |  |  |  |  |  |  |  | 1 |
| O130:H12 |  |  |  |  |  |  |  | 1 |  |  | 1 |  | O136:H16 | 1 |  |  |  | 1 |  |  |  |  |  |  |  | 2 |
| O152:H38 |  |  |  |  |  |  |  | 1 |  |  | 1 |  | O126:H8 |  |  | 1 |  |  |  |  |  |  |  |  |  | 1 |
| No. serotypes | 7 | 4 | 4 | 4 | 7 | 5 | 9 | 8 | 4 | 7 | **104** |  | O130:H? |  |  |  |  |  |  |  | 1 |  |  |  |  | 1 |
|  |  |  |  |  |  |  |  |  |  |  |  |  | O139:H? |  |  |  |  | 1 |  |  |  |  |  |  |  | 1 |
|  |  |  |  |  |  |  |  |  |  |  |  |  | No. serotypes | 5 | 4 | 6 | 3 | 6 | 3 | 3 | 6 | 3 | 3 | 4 | 2 | **96** |
|  |  |  |  |  |  |  |  |  |  |  |  |  |  |  |  |  |  |  |  |  |  |  |  |  |  |  |
| * Significantly higher prevalence than highlighted cow ID (pairwise Fisher's Exact, p < 0.05, Bonferroni correction) | | | | | | | | | | | | | | | | | | |  |  |  |  |  |  |  |  |
| **Bold**: top prevalent serotypes in each year; isolated > 5 samples | | | | | | | | | | | |  |  |  |  |  |  |  |  |  |  |  |  |  |  |  |

|  |  |  |  |  |  |  |  |  |  |  |  |  |  |  |  |  |  |
| --- | --- | --- | --- | --- | --- | --- | --- | --- | --- | --- | --- | --- | --- | --- | --- | --- | --- |
|  |  |  |  |  |  |  |  |  |  |  |  |  |  |  |  |  |  |
|  | **Year 3 Cow ID** | | | | | | | | | | | | | | | |  |
| **Serotype** | 21308 | 21309 | 21311 | 21314 | 21315 | 21317 | 21318 | 21323 | 21327 | 21328 | 21329 | 21331 | 21333 | 21334 | 21335 | 21341 |  |
| No. isolates | 13 | 13 | 4 | 6 | 12 | 11 | 7 | 8 | 10 | 5 | 6 | 10 | 9 | 8 | 8 | 7 | **SUM** |
| **O139:H19** | 1 | 1 | 2 | 4 | 10* | 5 | 2 | 5 |  | 1 | 4 | 7 | 1 |  | 1 | 4 | 38 |
| **O22:H8** |  | 3 |  |  |  | 2 | 4 | 1 | 2 |  |  |  |  | 5 | 5 |  | 22 |
| **O?(O108):H8** | 1 | 7 |  |  |  |  | 1 |  | 1 | 4 |  | 1 |  |  |  | 1 | 16 |
| **O6:H34** | 4 |  |  |  | 1 |  |  |  |  |  |  | 1 |  |  |  | 1 | 7 |
| **O91:H21** | 5 |  | 2 |  |  | 2 |  |  | 3 |  | 1 |  | 1 | 2 | 2 |  | 18 |
| **O113:H21** |  |  |  | 1 |  | 2 |  |  | 2 |  | 1 |  | 6 |  |  | 1 | 13 |
| O28ac:H25 |  |  |  |  |  |  |  | 1 | 2 |  |  |  |  |  |  |  | 3 |
| O84:H2 |  |  |  |  |  |  |  | 1 |  |  |  | 1 |  |  |  |  | 2 |
| O93:H28 | 1 |  |  |  |  |  |  |  |  |  |  |  |  |  |  |  | 1 |
| O111:NM | 1 |  |  |  |  |  |  |  |  |  |  |  |  |  |  |  | 1 |
| O137:H41 |  | 1 |  |  |  |  |  |  |  |  |  |  |  | 1 |  |  | 2 |
| OR:H8 |  | 1 |  | 1 |  |  |  |  |  |  |  |  |  |  |  |  | 2 |
| O137:H5 |  |  |  |  | 1 |  |  |  |  |  |  |  |  |  |  |  | 1 |
| OR:H21 |  |  |  |  |  |  |  |  |  |  |  |  | 1 |  |  |  | 1 |
| No. serotypes | 6 | 5 | 2 | 3 | 3 | 4 | 3 | 4 | 5 | 2 | 3 | 4 | 4 | 3 | 3 | 4 | **127** |
