# Supplementary material for: Multi-Year Persistence of Verotoxigenic Escherichia coli (VTEC) in a Closed Canadian Beef Herd: A Cohort Study
Source: Front Microbiol. 2018 Aug 31;9:2040. doi: 10.3389/fmicb.2018.02040 (PMC6127291; doi:10.3389/fmicb.2018.02040)
Supplement: Supplementary file 7 [file Table_7.DOCX]

| Supplementary Table 7. Frequency of virulotypes among top prevalent and top clinically associated | | | | | | | | | | | | | |
| --- | --- | --- | --- | --- | --- | --- | --- | --- | --- | --- | --- | --- | --- |
| serotypes among heifer isolates. | | |  |  |  |  |  |  |  |  |  |  |  |
|  |  |  |  |  |  |  |  |  |  |  |  |  |  |
|  |  | **Virulence Genes** | | | |  |  |  |  | ***vt2* subtype** | | |  |
| **Top Prevalent Serotypes (> 10 isolates)** | **No. total isolates** | ***vt1*** | ***vt2*** | ***hlyA*** | ***eae*A** | ***saa*** | **No. strains** |  | **No. total isolates** | ***vt2a*** | ***vt2c*** | ***vt2d*** | **No. strains** |
| O139:H19 | 96 | + | + | + | - | + | 95 |  | 91 | + | - | + | 83 |
|  |  | + | + | + | - | - | 1 |  |  | + | - | - | 6 |
|  |  |  |  |  |  |  |  |  |  | + | + | - | 1 |
|  |  |  |  |  |  |  |  |  |  | - | - | - | 1 |
| O22:H8 | 41 | + | - | + | - | + | 24 |  | 17 | - | + | - | 16 |
|  |  | + | + | + | - | + | 17 |  |  | - | - | - | 1 |
| O?(O108):H8^1^ | 40 | + | + | + | - | + | 40 |  | 38 | - | + | - | 25 |
|  |  |  |  |  |  |  |  |  |  | + | + | - | 12 |
|  |  |  |  |  |  |  |  |  |  | - | + | + | 1 |
| O130:H38 | 32 | + | + | + | - | + | 32 |  | 28 | + | - | + | 28 |
| O6:H34 | 26 | - | + | - | - | - | 26 |  | 24 | - | + | - | 18 |
|  |  |  |  |  |  |  |  |  |  | - | - | + | 6 |
| O91:H21 | 20 | + | + | + | - | + | 20 |  | 21 | + | + | + | 15 |
|  |  |  |  |  |  |  |  |  |  | + | - | - | 3 |
|  |  |  |  |  |  |  |  |  |  | + | - | + | 2 |
|  |  |  |  |  |  |  |  |  |  | - | - | - | 1 |
| O113:H21 | 16 | - | + | + | - | + | 14 |  | 16 | + | + | - | 9 |
|  |  | - | + | - | - | - | 2 |  |  | + | - | - | 4 |
|  |  |  |  |  |  |  |  |  |  | - | - | + | 2 |
|  |  |  |  |  |  |  |  |  |  | + | + | + | 1 |
| O28ac:H25 | 11 | - | + | + | - | + | 11 |  | 11 | + | + | + | 11 |
| **Top Pathogenic Serotypes** |  |  |  |  |  |  |  |  |  |  |  |  |  |
| O157:H7 | 4 | + | + | + | + | - | 4 |  | 4 | + | - | **-** | 4 |
| O26:H11 | 3 | + | - | + | + | - | 3 |  |  | *vt2* negative | | |  |
| O111:NM | 2 | + | - | + | + | - | 2 |  |  | *vt2* negative | | |  |
| **Other *eaeA+* Serotypes** |  |  |  |  |  |  |  |  |  |  |  |  |  |
| O84:H2 | 2 | + | - | + | + | - | 2 |  |  | *vt2* negative | | |  |
| O182:H25 | 2 | - | + | + | + | - | 2 |  | 2 | + | - | - | 2 |
|  |  |  |  |  |  |  |  |  |  |  |  |  |  |
| ^1^ All strains confirmed by *in silico* serotyping (n=14) were serotype O108:H8 | | | | | | | |  |  |  |  |  |  |
