# Supplementary material for: Multi-Year Persistence of Verotoxigenic Escherichia coli (VTEC) in a Closed Canadian Beef Herd: A Cohort Study
Source: Front Microbiol. 2018 Aug 31;9:2040. doi: 10.3389/fmicb.2018.02040 (PMC6127291; doi:10.3389/fmicb.2018.02040)
Supplement: Supplementary file 8 [file Table_8.DOCX]

| Supplementary Table 8. Prevalence of *vt1*, *vt2*, *vt1 vt2*, *hlyA, eaeA* and *saa* among SPT A-E isolates from heifers. | | | | | | | |
| --- | --- | --- | --- | --- | --- | --- | --- |
|  |  |  |  |  |  |  |  |
|  | **Seropathotype (SPT)** | | | | | |  |
|  | **SPT A** | **SPT B** | **SPT C** | **SPT D** | **SPT E** | **Unassigned** |  |
| **no. isolates** | **4** | **5** | **81** | **56** | **138** | **52** |  |
| ***vt1+ only*** | - | 100.0% | 30.9% | 5.4% | 1.4% | 1.9% |  |
| ***vt2+ only*** | - | - | 21.0% | 83.9% | 2.2% | 5.8% |  |
| ***vt1+ vt2+*** | 100.0% | - | 48.1% | 12.5% | 97.8% | 92.3% |  |
| ***hlyA+*** | 100.0% | 100.0% | 96.3% | 39.3% | 100.0% | 100.0% |  |
| ***eae*A*+*** | 100.0% | 100.0% | - | 7.1% | 0.0% | 0.0% |  |
| ***saa*+** | - | - | 96.3% | 26.8% | 97.8% | 96.2% |  |
|  |  |  |  |  |  |  |  |
| Unassigned - serotypes not previously identified as VTEC or strains not fully typed (O or H untypeable) | | | | | | |  |
| - negative in all isolates | |  |  |  |  |  |  |
