# Supplementary material for: Multi-Year Persistence of Verotoxigenic Escherichia coli (VTEC) in a Closed Canadian Beef Herd: A Cohort Study
Source: Front Microbiol. 2018 Aug 31;9:2040. doi: 10.3389/fmicb.2018.02040 (PMC6127291; doi:10.3389/fmicb.2018.02040)
Supplement: Supplementary file 9 [file Table_9.DOCX]

| Supplementary Table 9: Statistics source data. | | | | |
| --- | --- | --- | --- | --- |
|  |  |  |  |  |
| **Figure 2.** |  |  |  |  |
| Serotype | 2012-2013 | 2013-2014 | 2014-2015 |  |
| n = | 103 | 96 | 137 |  |
| O139:H19 | 18 | 30 | 48 |  |
| O22:H8 | 10 | 9 | 22 |  |
| O?(O108):H8 | 15 | 9 | 16 |  |
| O130:H38 | 19 | 13 | 0 |  |
| O6:H34 | 15 | 4 | 7 |  |
| O91:H21 | 2 | 1 | 18 |  |
| O113:H21 | 3 | 0 | 13 |  |
| O28ac:H25 | 4 | 4 | 3 |  |
|  |  |  |  |  |
| Serotype | Spring | Summer | Fall | Winter |
| n = | 75 | 88 | 103 | 70 |
| O139:H19 | 27 | 21 | 29 | 19 |
| O22:H8 | 3 | 12 | 15 | 11 |
| O?(O108):H8 | 10 | 8 | 11 | 11 |
| O130:H38 | 5 | 6 | 13 | 8 |
| O6:H34 | 7 | 5 | 8 | 6 |
| O91:H21 | 6 | 9 | 4 | 2 |
| O113:H21 | 5 | 2 | 4 | 5 |
| O28ac:H25 | 1 | 2 | 6 | 2 |
