# Supplementary material for: Multi-Year Persistence of Verotoxigenic Escherichia coli (VTEC) in a Closed Canadian Beef Herd: A Cohort Study
Source: Front Microbiol. 2018 Aug 31;9:2040. doi: 10.3389/fmicb.2018.02040 (PMC6127291; doi:10.3389/fmicb.2018.02040)
Supplement: Supplementary file 10 [file Table_10.DOCX]

| Supplementary Table 10. VTEC strains selected for whole genome sequencing. | | | | | | | |
| --- | --- | --- | --- | --- | --- | --- | --- |
|  |  |  |  |  |  |  |  |
| Strain | Serotype | Cohort-Year | Strain source | Isolation source | Isolation year | Isolation location | vf_amr dataset (n = 115) |
| ECI-2644 | O139:H19 | 1 | This study | Cow | 2012 | Canada | yes |
| ECI-2645 | O26:H11 | 1 | This study | Cow | 2012 | Canada | yes |
| ECI-2646 | O130:H38 | 1 | This study | Cow | 2012 | Canada | yes |
| ECI-2648 | O93:H28 | 1 | This study | Cow | 2012 | Canada | yes |
| ECI-2659 | O108:H8 | 1 | This study | Cow | 2012 | Canada | yes |
| ECI-2663 | O26:H11 | 1 | This study | Cow | 2012 | Canada | yes |
| ECI-2665 | O130:H38 | 1 | This study | Cow | 2012 | Canada |  |
| ECI-2667 | O6:H34 | 1 | This study | Cow | 2012 | Canada | yes |
| ECI-2784 | O139:H19 | 1 | This study | Cow | 2012 | Canada |  |
| ECI-2785 | O130:H38 | 1 | This study | Cow | 2012 | Canada |  |
| ECI-2789 | O182:H25 | 1 | This study | Cow | 2012 | Canada | yes |
| ECI-2790 | O6:H34 | 1 | This study | Cow | 2012 | Canada |  |
| ECI-2792 | O139:H19 | 1 | This study | Cow | 2012 | Canada |  |
| ECI-2864 | O108:H8 | 1 | This study | Cow | 2012 | Canada | yes |
| ECI-2865 | O132:H18 | 1 | This study | Cow | 2012 | Canada | yes |
| ECI-2866 | O22:H8 | 1 | This study | Cow | 2012 | Canada | yes |
| ECI-2867 | O139:H19 | 1 | This study | Cow | 2012 | Canada |  |
| ECI-2868 | O22:H8 | 1 | This study | Cow | 2012 | Canada |  |
| ECI-2872 | O130:H38 | 1 | This study | Cow | 2012 | Canada | yes |
| ECI-2877 | O22:H8 | 1 | This study | Cow | 2012 | Canada |  |
| ECI-2918 | O132:H18 | 1 | This study | Cow | 2012 | Canada | yes |
| ECI-2927 | O108:H8 | 1 | This study | Cow | 2012 | Canada |  |
| ECI-2929 | O2:H6 | 1 | This study | Cow | 2012 | Canada | yes |
| ECI-2941 | O6:H34 | 1 | This study | Cow | 2012 | Canada |  |
| ECI-2944 | O22:H8 | 1 | This study | Cow | 2012 | Canada |  |
| ECI-2960 | O139:H19 | 1 | This study | Cow | 2012 | Canada |  |
| ECI-2961 | O139:H19 | 1 | This study | Cow | 2012 | Canada |  |
| ECI-2964 | O28ac/O42:H25 | 1 | This study | Cow | 2012 | Canada | yes |
| ECI-2972 | O6:H34 | 1 | This study | Cow | 2012 | Canada | yes |
| ECI-2975 | O22:H8 | 1 | This study | Cow | 2012 | Canada |  |
| ECI-3013 | O132:H18 | 1 | This study | Cow | 2012 | Canada | yes |
| ECI-3014 | O26:H11 | 1 | This study | Cow | 2012 | Canada | yes |
| ECI-3016 | O113:H21 | 1 | This study | Cow | 2012 | Canada | yes |
| ECI-3019 | O108:H8 | 1 | This study | Cow | 2012 | Canada |  |
| ECI-3020 | O139:H19 | 1 | This study | Cow | 2012 | Canada |  |
| ECI-3022 | O6:H34 | 1 | This study | Cow | 2012 | Canada |  |
| ECI-3024 | O132:H18 | 1 | This study | Cow | 2012 | Canada | yes |
| ECI-3073 | O139:H19 | 1 | This study | Cow | 2012 | Canada |  |
| ECI-3074 | O6:H34 | 1 | This study | Cow | 2012 | Canada |  |
| ECI-3075 | O6:H34 | 1 | This study | Cow | 2012 | Canada | yes |
| ECI-3078 | O130:H38 | 1 | This study | Cow | 2012 | Canada |  |
| ECI-3080 | O139:H19 | 1 | This study | Cow | 2012 | Canada | yes |
| ECI-3083 | O108:H8 | 1 | This study | Cow | 2012 | Canada |  |
| ECI-3084 | O108:H8 | 1 | This study | Cow | 2012 | Canada |  |
| ECI-3085 | O139:H19 | 1 | This study | Cow | 2012 | Canada |  |
| ECI-3086 | O28ac/O42:H25 | 1 | This study | Cow | 2012 | Canada | yes |
| ECI-3088 | O182:H25 | 1 | This study | Cow | 2012 | Canada | yes |
| ECI-3089 | O130:H38 | 1 | This study | Cow | 2012 | Canada |  |
| ECI-3091 | O130:H38 | 1 | This study | Cow | 2012 | Canada | yes |
| ECI-3096 | O108:H8 | 1 | This study | Cow | 2012 | Canada |  |
| ECI-3100 | O6:H34 | 1 | This study | Cow | 2012 | Canada |  |
| ECI-3101 | O130:H38 | 1 | This study | Cow | 2012 | Canada |  |
| ECI-3102 | O6:H34 | 1 | This study | Cow | 2012 | Canada |  |
| ECI-3108 | O130:H38 | 1 | This study | Cow | 2012 | Canada |  |
| ECI-3109 | O139:H19 | 1 | This study | Cow | 2012 | Canada |  |
| ECI-3110 | O108:H8 | 1 | This study | Cow | 2012 | Canada | yes |
| ECI-3111 | O6:H34 | 1 | This study | Cow | 2012 | Canada | yes |
| ECI-3112 | O139:H19 | 1 | This study | Cow | 2012 | Canada |  |
| ECI-3113 | O139:H19 | 1 | This study | Cow | 2012 | Canada |  |
| ECI-3114 | O130:H38 | 1 | This study | Cow | 2012 | Canada | yes |
| ECI-3116 | O130:H38 | 1 | This study | Cow | 2012 | Canada |  |
| ECI-3119 | O130:H38 | 1 | This study | Cow | 2012 | Canada |  |
| ECI-3123 | O130:H38 | 1 | This study | Cow | 2012 | Canada | yes |
| ECI-3124 | O108:H8 | 1 | This study | Cow | 2012 | Canada |  |
| ECI-3125 | O134/O46:H38 | 1 | This study | Cow | 2012 | Canada | yes |
| ECI-3127 | O22:H8 | 1 | This study | Cow | 2012 | Canada |  |
| ECI-3152 | O6:H34 | 1 | This study | Cow | 2013 | Canada |  |
| ECI-3158 | O108:H8 | 1 | This study | Cow | 2013 | Canada |  |
| ECI-3175 | O22:H8 | 1 | This study | Cow | 2013 | Canada |  |
| ECI-3186 | O130:H38 | 1 | This study | Cow | 2013 | Canada |  |
| ECI-3188 | O108:H8 | 1 | This study | Cow | 2013 | Canada |  |
| ECI-3192 | O130:H38 | 1 | This study | Cow | 2013 | Canada |  |
| ECI-3196 | O22:H8 | 1 | This study | Cow | 2013 | Canada |  |
| ECI-3200 | O6:H34 | 1 | This study | Cow | 2013 | Canada | yes |
| ECI-3219 | O108:H8 | 1 | This study | Cow | 2013 | Canada |  |
| ECI-3229 | O139:H19 | 1 | This study | Cow | 2013 | Canada |  |
| ECI-3242 | O130:H38 | 1 | This study | Cow | 2013 | Canada | yes |
| ECI-3250 | O139:H19 | 1 | This study | Cow | 2013 | Canada |  |
| ECI-3251 | O28ac/O42:H25 | 1 | This study | Cow | 2013 | Canada | yes |
| ECI-3269 | O139:H19 | 1 | This study | Cow | 2013 | Canada |  |
| ECI-3279 | O108:H8 | 1 | This study | Cow | 2013 | Canada |  |
| ECI-3280 | O139:H19 | 1 | This study | Cow | 2013 | Canada |  |
| ECI-3308 | O130:H38 | 1 | This study | Cow | 2013 | Canada |  |
| ECI-3335 | O108:H8 | 1 | This study | Cow | 2013 | Canada | yes |
| ECI-3359 | O6:H34 | 1 | This study | Cow | 2013 | Canada | yes |
| ECI-3360 | O22:H8 | 1 | This study | Cow | 2013 | Canada | yes |
| ECI-3388 | O91:H21 | 1 | This study | Cow | 2013 | Canada | yes |
| ECI-3389 | O130:H38 | 1 | This study | Cow | 2013 | Canada |  |
| ECI-3392 | O113:H21 | 1 | This study | Cow | 2013 | Canada | yes |
| ECI-3393 | O139:H19 | 1 | This study | Cow | 2013 | Canada |  |
| ECI-3407 | O91:H21 | 1 | This study | Cow | 2013 | Canada | yes |
| ECI-3458 | O130:H38 | 1 | This study | Cow | 2013 | Canada |  |
| ECI-3462 | O108:H8 | 2 | This study | Cow | 2013 | Canada | yes |
| ECI-3513 | O139:H19 | 2 | This study | Cow | 2013 | Canada | yes |
| ECI-3548 | O111:NM/H8 | 2 | This study | Cow | 2013 | Canada | yes |
| ECI-3553 | O139:H19 | 2 | This study | Cow | 2013 | Canada |  |
| ECI-3565 | O139:H19 | 2 | This study | Cow | 2013 | Canada |  |
| ECI-3567 | O22:H8 | 2 | This study | Cow | 2013 | Canada | yes |
| ECI-3602 | O22:H8 | 2 | This study | Cow | 2013 | Canada |  |
| ECI-3614 | O134/O46:H38 | 2 | This study | Cow | 2013 | Canada | yes |
| ECI-3615 | O157:H7 | 2 | This study | Cow | 2013 | Canada | yes |
| ECI-3616 | O139:H19 | 2 | This study | Cow | 2013 | Canada |  |
| ECI-3622 | O157:H7 | 2 | This study | Cow | 2013 | Canada | yes |
| ECI-3632 | O157:H7 | 2 | This study | Cow | 2013 | Canada | yes |
| ECI-3645 | O139:H19 | 2 | This study | Cow | 2013 | Canada |  |
| ECI-3647 | O43:H2 | 2 | This study | Cow | 2013 | Canada | yes |
| ECI-3648 | O130:H11 | 2 | This study | Cow | 2013 | Canada | yes |
| ECI-3650 | O28ac/O42:H25 | 2 | This study | Cow | 2013 | Canada | yes |
| ECI-3651 | O136:H16 | 2 | This study | Cow | 2013 | Canada | yes |
| ECI-3653 | O136:H16 | 2 | This study | Cow | 2013 | Canada | yes |
| ECI-3668 | O91:H21 | 2 | This study | Cow | 2013 | Canada | yes |
| ECI-3670 | O126:H8 | 2 | This study | Cow | 2013 | Canada | yes |
| ECI-3674 | O22:H8 | 2 | This study | Cow | 2013 | Canada |  |
| ECI-3681 | O139:H19 | 2 | This study | Cow | 2013 | Canada |  |
| ECI-3683 | O43:H2 | 2 | This study | Cow | 2013 | Canada | yes |
| ECI-3684 | O43:H2 | 2 | This study | Cow | 2013 | Canada | yes |
| ECI-3688 | O22:H8 | 2 | This study | Cow | 2013 | Canada |  |
| ECI-3708 | O22:H8 | 2 | This study | Cow | 2013 | Canada |  |
| ECI-3710 | O157:H7 | 2 | This study | Cow | 2013 | Canada | yes |
| ECI-3711 | O139:H19 | 2 | This study | Cow | 2013 | Canada | yes |
| ECI-3717 | O130:H38 | 2 | This study | Cow | 2013 | Canada | yes |
| ECI-3733 | O139:H19 | 2 | This study | Cow | 2013 | Canada |  |
| ECI-3807 | O139:H19 | 2 | This study | Cow | 2013 | Canada |  |
| ECI-3811 | O22:H8 | 2 | This study | Cow | 2013 | Canada | yes |
| ECI-3841 | O139:H19 | 2 | This study | Cow | 2014 | Canada |  |
| ECI-3860 | O139:H19 | 2 | This study | Cow | 2014 | Canada |  |
| ECI-3886 | O91:H21 | 3 | This study | Cow | 2014 | Canada | yes |
| ECI-3889 | O108:H8 | 3 | This study | Cow | 2014 | Canada | yes |
| ECI-3894 | O137:H41 | 3 | This study | Cow | 2014 | Canada | yes |
| ECI-3913 | O91:H21 | 3 | This study | Cow | 2014 | Canada |  |
| ECI-3921 | O91:H21 | 3 | This study | Cow | 2014 | Canada |  |
| ECI-3927 | O113:H21 | 3 | This study | Cow | 2014 | Canada | yes |
| ECI-3929 | O139:H19 | 3 | This study | Cow | 2014 | Canada | yes |
| ECI-3930 | O139:H19 | 3 | This study | Cow | 2014 | Canada |  |
| ECI-3949 | O91:H21 | 3 | This study | Cow | 2014 | Canada |  |
| ECI-3951 | O139:H19 | 3 | This study | Cow | 2014 | Canada |  |
| ECI-3955 | O22:H8 | 3 | This study | Cow | 2014 | Canada | yes |
| ECI-3963 | O91:H21 | 3 | This study | Cow | 2014 | Canada |  |
| ECI-3965 | O137:H41 | 3 | This study | Cow | 2014 | Canada | yes |
| ECI-3968 | O91:H21 | 3 | This study | Cow | 2014 | Canada |  |
| ECI-3971 | O91:H21 | 3 | This study | Cow | 2014 | Canada |  |
| ECI-4008 | O91:H21 | 3 | This study | Cow | 2014 | Canada |  |
| ECI-4015 | O22:H8 | 3 | This study | Cow | 2014 | Canada |  |
| ECI-4016 | O91:H21 | 3 | This study | Cow | 2014 | Canada | yes |
| ECI-4020 | O139:H19 | 3 | This study | Cow | 2014 | Canada |  |
| ECI-4021 | O84:H2 | 3 | This study | Cow | 2014 | Canada | yes |
| ECI-4023 | O22:H8 | 3 | This study | Cow | 2014 | Canada |  |
| ECI-4029 | O91:H21 | 3 | This study | Cow | 2014 | Canada |  |
| ECI-4130 | O91:H21 | 3 | This study | Cow | 2014 | Canada |  |
| ECI-4131 | O139:H19 | 3 | This study | Cow | 2014 | Canada |  |
| ECI-4141 | O139:H19 | 3 | This study | Cow | 2014 | Canada |  |
| ECI-4246 | O91:H21 | 3 | This study | Cow | 2014 | Canada |  |
| ECI-4264 | O22:H8 | 3 | This study | Cow | 2014 | Canada |  |
| ECI-4276 | O139:H19 | 3 | This study | Cow | 2014 | Canada |  |
| ECI-4286 | O22:H8 | 3 | This study | Cow | 2014 | Canada |  |
| ECI-4290 | O22:H8 | 3 | This study | Cow | 2014 | Canada |  |
| ECI-4294 | O139:H19 | 3 | This study | Cow | 2014 | Canada |  |
| ECI-4319 | O91:H21 | 3 | This study | Cow | 2014 | Canada |  |
| ECI-4324 | O22:H8 | 3 | This study | Cow | 2014 | Canada |  |
| ECI-4351 | O139:H19 | 3 | This study | Cow | 2014 | Canada |  |
| ECI-4355 | O22:H8 | 3 | This study | Cow | 2014 | Canada |  |
| ECI-4374 | O91:H21 | 3 | This study | Cow | 2014 | Canada |  |
| ECI-4380 | O139:H19 | 3 | This study | Cow | 2014 | Canada |  |
| ECI-4384 | O91:H21 | 3 | This study | Cow | 2014 | Canada |  |
| ECI-4407 | O22:H8 | 3 | This study | Cow | 2014 | Canada |  |
| ECI-4412 | O22:H8 | 3 | This study | Cow | 2014 | Canada |  |
| ECI-4487 | O139:H19 | 3 | This study | Cow | 2014 | Canada |  |
| ECI-4505 | O91:H21 | 3 | This study | Cow | 2014 | Canada |  |
| ECI-4509 | O22:H8 | 3 | This study | Cow | 2014 | Canada |  |
| ECI-4523 | O22:H8 | 3 | This study | Cow | 2014 | Canada |  |
| ECI-4528 | O22:H8 | 3 | This study | Cow | 2014 | Canada |  |
| ECI-4536 | O22:H8 | 3 | This study | Cow | 2015 | Canada |  |
| ECI-4545 | O139:H19 | 3 | This study | Cow | 2015 | Canada |  |
| ECI-4546 | O22:H8 | 3 | This study | Cow | 2015 | Canada | yes |
| ECI-4561 | O91:H21 | 3 | This study | Cow | 2015 | Canada |  |
| ECI-4568 | O139:H19 | 3 | This study | Cow | 2015 | Canada |  |
| ECI-4616 | O139:H19 | 3 | This study | Cow | 2015 | Canada |  |
| ECI-4620 | O139:H19 | 3 | This study | Cow | 2015 | Canada | yes |
| ECI-4622 | O91:H21 | 3 | This study | Cow | 2015 | Canada | yes |
| DRR103561 | O26:H11 | n/a | NCBI-SRA | Human | 2011 | Japan |  |
| ERR1010207 | O134/O46:H38 | n/a | NCBI-SRA | Cow | 2008 | France | yes |
| ERR434552 | O91:H21 | n/a | NCBI-SRA | Human | 2010 | UK |  |
| ERR439599 | O139:H19 | n/a | NCBI-SRA | Human | 2003 | UK | yes |
| ERR439644 | O2:H6 | n/a | NCBI-SRA | Human | 2002 | UK | yes |
| SRR1580930 | O157:H7 | n/a | NCBI-SRA | Human | 2014 | USA | yes |
| SRR1758758 | O157:H7 | n/a | NCBI-SRA | Human | 2014 | USA |  |
| SRR1767918 | O28ac/O42:H25 | n/a | NCBI-SRA | Human | n/a | USA | yes |
| SRR1999112 | O130:H11 | n/a | NCBI-SRA | Human | 2012 | USA | yes |
| SRR2002646 | O113:H21 | n/a | NCBI-SRA | Human | 2015 | USA | yes |
| SRR2014851 | O91:H21 | n/a | NCBI-SRA | Human | 2009 | USA |  |
| SRR2038679 | O6:H34 | n/a | NCBI-SRA | Human | 2015 | USA | yes |
| SRR2038687 | O157:H7 | n/a | NCBI-SRA | Human | 2015 | USA |  |
| SRR2480634 | O126:H8 | n/a | NCBI-SRA | Human | n/a | USA | yes |
| SRR2481230 | O136:H16 | n/a | NCBI-SRA | Human | n/a | USA | yes |
| SRR2562687 | O130:H11 | n/a | NCBI-SRA | Human | 2014 | USA | yes |
| SRR2775032 | O111:NM/H8 | n/a | NCBI-SRA | Cow | 2015 | USA | yes |
| SRR3113785 | O126:H8 | n/a | NCBI-SRA | Human | 2015 | USA | yes |
| SRR3133016 | O22:H8 | n/a | NCBI-SRA | Kale | 2015 | USA |  |
| SRR3290219 | O84:H2 | n/a | NCBI-SRA | Human | 2015 | USA | yes |
| SRR3457715 | O2:H6 | n/a | NCBI-SRA | Mouse | 1986 | USA |  |
| SRR3574228 | O91:H21 | n/a | NCBI-SRA | Human | 2016 | UK | yes |
| SRR3574264 | O91:H21 | n/a | NCBI-SRA | Human | 2016 | UK |  |
| SRR3574266 | O93:H28 | n/a | NCBI-SRA | Human | 2016 | UK | yes |
| SRR3574336 | O91:H21 | n/a | NCBI-SRA | Human | 2016 | UK |  |
| SRR3578167 | O43:H2 | n/a | NCBI-SRA | Human | 2015 | UK |  |
| SRR3578976 | O43:H2 | n/a | NCBI-SRA | Human | 2015 | UK | yes |
| SRR3581387 | O182:H25 | n/a | NCBI-SRA | Human | 2015 | UK | yes |
| SRR3581498 | O2:H6 | n/a | NCBI-SRA | Human | 2015 | UK | yes |
| SRR3883023 | O111:NM/H8 | n/a | NCBI-SRA | Human | 2016 | USA | yes |
| SRR3929485 | O22:H8 | n/a | NCBI-SRA | Tomato | 2003 | USA | yes |
| SRR3929488 | O136:H16 | n/a | NCBI-SRA | Spinach | 2008 | USA | yes |
| SRR3929503 | O28ac/O42:H25 | n/a | NCBI-SRA | Parsley | 2005 | USA | yes |
| SRR3931168 | O43:H2 | n/a | NCBI-SRA | Cilantro | 2013 | USA | yes |
| SRR3931188 | O28ac/O42:H25 | n/a | NCBI-SRA | Spinach | 2009 | USA | yes |
| SRR3931195 | O134/O46:H38 | n/a | NCBI-SRA | Food | 2010 | USA | yes |
| SRR3931237 | O130:H11 | n/a | NCBI-SRA | Cantalope | 2011 | USA | yes |
| SRR3931256 | O26:H11 | n/a | NCBI-SRA | Spinach | 2012 | USA | yes |
| SRR3931266 | O113:H21 | n/a | NCBI-SRA | Spinach | 2009 | USA | yes |
| SRR3931267 | O91:H21 | n/a | NCBI-SRA | Spinach | 2012 | USA |  |
| SRR3931276 | O130:H11 | n/a | NCBI-SRA | Basil | 2013 | USA |  |
| SRR3951609 | O130:H11 | n/a | NCBI-SRA | Clover Sprout | 2014 | USA |  |
| SRR3987538 | O91:H21 | n/a | NCBI-SRA | Chicken | 1990 | USA |  |
| SRR3987702 | O2:H6 | n/a | NCBI-SRA | Cat | 1985 | USA | yes |
| SRR3989717 | O91:H21 | n/a | NCBI-SRA | Bird | 2012 | USA |  |
| SRR3989728 | O43:H2 | n/a | NCBI-SRA | Bird | 2012 | USA | yes |
| SRR3989729 | O43:H2 | n/a | NCBI-SRA | Bird | 2012 | USA |  |
| SRR4184713 | O22:H8 | n/a | NCBI-SRA | Human | 2015 | UK |  |
| SRR4195714 | O84:H2 | n/a | NCBI-SRA | Human | 2015 | UK |  |
| SRR4195774 | O84:H2 | n/a | NCBI-SRA | Human | 2016 | UK | yes |
| SRR4228456 | O113:H21 | n/a | NCBI-SRA | Human | 2016 | USA | yes |
| SRR4263660 | O22:H8 | n/a | NCBI-SRA | Human | 1987 | Canada | yes |
| SRR4340488 | O84:H2 | n/a | NCBI-SRA | Human | 1989 | USA |  |
| SRR4340511 | O113:H21 | n/a | NCBI-SRA | Human | n/a | USA |  |
| SRR4340527 | O91:H21 | n/a | NCBI-SRA | Sheep | 1992 | USA |  |
| SRR5187829 | O91:H21 | n/a | NCBI-SRA | Human | 1993 | USA | yes |
| SRR5318919 | O157:H7 | n/a | NCBI-SRA | Cow | 1999 | USA | yes |
| SRR5330851 | O113:H21 | n/a | NCBI-SRA | Food | n/a | USA |  |
| SRR5330856 | O91:H21 | n/a | NCBI-SRA | Pork | n/a | USA |  |
| SRR5330860 | O91:H21 | n/a | NCBI-SRA | Human | 1987 | Canada | yes |
| SRR5330929 | O111:NM/H8 | n/a | NCBI-SRA | Human | 2001 | USA |  |
| SRR5330936 | O84:H2 | n/a | NCBI-SRA | Cow | 1992 | USA | yes |
| SRR5683232 | O111:NM/H8 | n/a | NCBI-SRA | Beef | 2000 | USA |  |
| SRR6061339 | O91:H21 | n/a | NCBI-SRA | unknown | 2010 | Canada |  |
| SRR6061349 | O22:H8 | n/a | NCBI-SRA | unknown | 2014 | Canada |  |
| SRR6061353 | O22:H8 | n/a | NCBI-SRA | unknown | 2010 | Canada |  |
| SRR6197835 | O111:NM/H8 | n/a | NCBI-SRA | Beef | 2017 | USA | yes |
| SRR6321269 | O26:H11 | n/a | NCBI-SRA | Human | 2016 | UK | yes |
| SRR6321271 | O26:H11 | n/a | NCBI-SRA | Human | 2016 | UK | yes |
| SRR6321291 | O22:H8 | n/a | NCBI-SRA | Human | 2015 | UK | yes |
| SRR6373579 | O157:H7 | n/a | NCBI-SRA | Beef | n/a | USA | yes |
| SRR6373714 | O26:H11 | n/a | NCBI-SRA | Human | 2011 | USA |  |
